# Progressive accumulation of circulating CD27^−^CD28^−^ effector/memory CD8^+^ T cells in patients with lung cancer blunts responses to immune checkpoint inhibitor therapy

**DOI:** 10.1038/s12276-025-01448-7

**Published:** 2025-05-01

**Authors:** Sung-Woo Lee, Ju Sik Yun, Young Ju Kim, Saei Jeong, Jeong Eun Noh, Hee-Ok Kim, Hyun-Ju Cho, Cheol-Kyu Park, In-Jae Oh, Jae-Ho Cho

**Affiliations:** 1 Department of Microbiology and Immunology, Chonnam National University Medical School, Gwangju, Republic of Korea; 2 Medical Research Center for Combinatorial Tumor Immunotherapy, Chonnam National University Medical School, Gwangju, Republic of Korea; 3 Department of Thoracic and Cardiovascular Surgery, Chonnam National University Medical School, Hwasun Hospital, Gwangju, Republic of Korea; 4 National Immunotherapy Innovation Center, Chonnam National University Medical School, Gwangju, Republic of Korea; 5 BioMedical Sciences Graduate Program, Chonnam National University Medical School, Gwangju, Republic of Korea; 6 Selecxine Inc., Seoul, Republic of Korea; 7 Department of Internal Medicine, Chonnam National University Medical School, Hwasun Hospital, Gwangju, Republic of Korea

**Keywords:** Immunotherapy, Non-small-cell lung cancer, Lymphocyte differentiation, Predictive markers, CD8-positive T cells

## Abstract

Suppression of tumor-reactive CD8^+^ T cells is common within the tumor microenvironment. However, little is known about how tumors systemically affect the overall CD8^+^ T cell compartment. Here we demonstrate that peripheral blood CD8^+^ T cells from patients with lung cancer showed altered compositions particularly within CD45RA^−^CCR7^−^ effector memory subpopulation. Specifically, patients with lung cancer exhibited increased frequency of more differentiated effector memory cells, which are less susceptible to T cell-receptor-induced proliferation. Further analysis using single-cell RNA sequencing revealed that these alterations were correlated with reduced quiescence and increased spontaneous activation at a systemic level, indicative of homeostatic dysregulation of the entire CD8^+^ T cell population. This phenomenon was found to be correlated with a poor clinical response to immune checkpoint inhibitor therapy across four independent cohorts, consisting of a total of 224 patients with lung cancer. These findings suggest that lung cancers continue to counteract potentially tumor-reactive CD8^+^ T cells by inducing homeostatic dysregulation of the entire CD8^+^ T cell compartment systematically.

## Lung cancer alters CD8 T cell dynamics and function

The immune system can detect and fight cancer, but cancer can develop ways to resist this attack. This study analyzes blood samples from 349 patients with lung cancer to understand how lung cancer affects CD8^+^ T cells, key immune cells that help fight cancer. The results showed that these cells had unusual changes, making them less able to multiply and fight cancer effectively. These changes developed gradually across different types of CD8^+^ T cells, both those targeting tumors and those that did not. The study suggests that an imbalance in the immune system may be responsible. Furthermore, patients with these immune changes responded poorly to immune checkpoint inhibitor therapy. This highlights a new way cancer avoids immune detection by disrupting CD8^+^ T cell balance and suggests that understanding these changes could help improve cancer treatments.

This summary was initially drafted using artificial intelligence, then revised and fact-checked by the author.

## Introduction

It has long been recognized that the immune system can detect, monitor and eliminate cancer, and today, numerous studies have made this even more evident. For example, the correlation between an increase in tumor-infiltrating lymphocytes (especially CD8^+^ T cells) and responsiveness to immunotherapy is known to indicate a cancer-immune interaction^1–4^. In addition, various markers suggestive of an active antitumor immune response within tumor tissues (for example, PD-L1 expression, tumor mutation burden and interferon (IFN)-γ production) have been reported with their associations with clinical responses to immunotherapy^3–13^. However, paradoxically, the presence of antitumor immunity highlights the fact that cancer can evolve immune resistance as a protective adaptation against immune attack. Indeed, among various evasion mechanisms, IFN-γ produced by tumor-reactive CD8^+^ T cells is thought to inhibit antitumor activity by inducing PD-L1 expression within tumor tissues^14–16^. Immune checkpoint inhibitors (ICIs) targeting PD-1/PD-L1 inhibit such cancer-induced adaptive resistance mechanisms and, therefore, have revolutionized cancer treatment^4–7,17–19^. However, they still have substantial limitations in that they are only effective in ~20% of patients with cancer^20–22^. Furthermore, the fact that ~60% of nonresponders to anti-PD-(L)1 therapy belong to a group with high tumor PD-L1 expression^19^ suggests that there are much broader and more complex immune evasion mechanisms beyond immune checkpoint-mediated suppression in the tumor microenvironment (TME).

Tumor-specific CD8^+^ T cells have shown to be heterogeneous in their activation and differentiation status, with a correlation with clinical responses to ICI therapy^23–32^. Such heterogeneity is considered to reflect various contexts of antitumor immune responses, but the precise nature of these contexts (both within and outside of TME) and their possible relevance to cancer-associated immune resistance mechanisms are still poorly understood. Despite growing knowledge of the mechanisms of cancer-associated resistance to tumor-reactive CD8^+^ T cells and the clinical applications of this understanding^33,34^, the overall survival of patients with cancer has not markedly improved^35–37^. In this study, we seek to address the question of whether cancer can affect the immunosurveillance function of CD8^+^ T cells at a systemic level and explore its underlying mechanisms. We conducted an extensive retrospective analysis of peripheral blood CD8^+^ T cells from 349 patients with lung cancer and found an aberrantly gross alteration in the peripheral blood CD8^+^ T cell compartment, which showed accumulation of proliferation-incompetent effector subsets. This phenomenon strongly correlated with genetic and proteomic profiles indicative of homeostatic dysregulation of CD8^+^ T cells at a systemic level, leading to poor clinical responses to ICI therapy. Therefore, we uncovered a new evasion mechanism by which cancer avoids attack by potentially tumor-reactive CD8^+^ T cells.

## Materials and Methods

### Human samples

The peripheral blood of the patients was provided by the Biobank of Chonnam National University Hwasun Hospital, a member of the Korea Biobank Network. No clinicopathologic criteria such as sex, age or weight were considered for exclusion. All patients provided written informed consent in accordance with local regulations (South Korea). This study was approved by the Institutional Review Boards of Chonnam National University Medical School and Hwasun Hospital (CNUHH-2018-036 and CNUHH-2021-045). The patients were divided into four cohorts as follows: stage IV non-small cell lung cancer (NSCLC) who received ICIs as the second- and third-line (cohort 1) or the first-line treatment (cohort 2), extensive disease stage small cell lung cancer (SCLC) who received ICI plus chemotherapy as the first-line treatment (cohort 3) and stage III NSCLC who received concurrent chemoradiation therapy (CCRT) followed by ICI consolidation treatment (cohort 4). The pretreatment peripheral blood was collected 1−10 days before or on the initial date of ICI treatment or CCRT. ICIs were administered intravenously for 60 min every 3 weeks (cohorts 1, 2 and 3). For patients who completed CCRT without progression (cohort 4), ICI (durvalumab) was administered every 2 weeks. The post-treatment blood samples were collected 7−14 days after the first ICI treatment. In cohort 4, durvalumab consolidation treatment continued for a maximum duration of 12 months. Most of the blood from healthy individuals was acquired from the Korean Red Cross (Blood donors). Some of the healthy individuals were recruited at Chonnam National University Hwasun Hospital. The information about blood donors from the Korean Red Cross was not accessible due to local regulations. The written informed consent from these donors were formally exempted by the Institutional Review Board in accordance with the Korean Bioethics and Safety Act. The peripheral whole blood was collected from the cephalic vein using BD Vacutainer (BD) and immediately processed with Lymphoprep (Stemcell Technologies) to obtain peripheral blood mononuclear cells (PBMCs). The PBMCs were fast frozen at −80 °C using 10% dimethyl sulfoxide (Merck) in fetal bovine serum (Gibco), then thawed in a 37 °C water bath upon experiment.

### Clinical analysis

During ICI treatment, a follow-up computed tomography scan for the response assessment was performed every two to three cycles (cohorts 1, 2 and 3) or four to six cycles (cohort 4). The clinical response to the treatment was defined by the Response Evaluation Criteria in Solid Tumors (RECIST) version 1.1. The best response was defined as complete response if cancer was no longer detectable, partial response (PR) if the size of the cancer decreased, stable disease if there was no progression and progressive disease if the size of the cancer increased on computed tomography up to 3 months after starting the treatment. In addition, PR or stable disease by 6 months was defined as durable clinical benefit (DCB). The forrest plot was analyzed using the indicated variables against PR. The hazard ratios were calculated against one factor (reference) in each variable.

### Flow cytometry

The thawed PBMCs were used for flow cytometry. For intracellular molecules, the samples were fixed/permeabilized with BD Cytofix/Cytoperm (BD) or FOXP3/Transcription Factor Staining Buffer Set (eBioscience) before intracellular-staining. The samples were run using CytoFLEX S (Beckman Coulter) or CytoFLEX LX (Beckman Coulter) and analyzed using Flowjo software (Tree Star). The following antibodies for flow cytometry were purchased from Biolegend, BD or Invitrogen: anti-CD8α (RPA-T8), anti-CD4 (A161A1), anti-CD3 (OKT3), anti-CCR7 (G043H7), anti-CD45RA (HI100), anti-CD27 (LG.7F9), anti-CD28 (CD28.2), anti-IL-2 (MQ1-17H12), anti-CD127 (hIL-7R-M21), anti-CD45RO (UCHL1), anti-CXCR3 (G025H7), anti-TCF1 (7F11A10), anti-CD44 (BJ18), anti-CD69 (FN50), anti-PD-1 (J105), anti-CD95 (DX2), anti-CD103 (Ber-ACT8), anti-ICOS (C398.4A), anti-CD5 (L17F12), anti-KLRG1 (14C2A07), anti-CD56 (5.1H11), anti-Bcl2 (100), anti-CD122 (TU27), anti-Ki-67 (Ki-67), anti-CD62L (DREG-56), anti-CD40L (24-31), anti-IFN-γ (B27), anti-CD39 (A1), anti-Perforin (B-D48), anti-Granzyme B (GB11), anti-CD57 (HCD57), anti-CXCR4 (12G5), anti-41BB (4B4-1), anti-TNF (MAb11), anti-T-bet (4B10), anti-Granzyme K (G3H69), anti-CX3CR1 (2A9-1) and anti-CD73 (AD2). In some experiments, fluorochrome-conjugated monoclonal antibodies (for CD8, CD27 and CD45RA) generated by SELECXINE were used.

### Patient grouping strategies

To investigate biomarker efficiency of CD27^−^CD28^−^-Tem and CD27^+^CD28^+^-Temra, CD27^−^CD28^−^-Tem and CD27^+^CD28^+^-Temra frequencies were assessed in total Tem and total Temra, respectively, in the peripheral blood of patients with lung cancer and healthy individuals. The patients with CD27^−^CD28^−^-Tem and CD27^+^CD28^+^-Temra frequencies higher than that of most (>~80%) healthy individuals who were analyzed under the same experiment settings (CD27^−^CD28^−^-Tem ≥~36% and CD27^+^CD28^+^-Temra ≥~17.2% for patients with NSCLC and CD27^−^CD28^−^-Tem ≥~42% and CD27^+^CD28^+^-Temra ≥~11% for patients with SCLC) were considered CD27^−^CD28^−^-Tem^hi^ and CD27^+^CD28^+^-Temra^hi^.

### In vitro T cell proliferation

Anti-CD3 (clone OKT3) and anti-CD28 (clone CD28.2) antibodies were coated onto a flat-bottom 96-well immuno-plate (Thermo Fisher Scientific) overnight at 4 °C. DP-Tem and DN-Tem were purified using CytoFLEX SRT (Beckman Coulter), then labeled with CellTrace Violet (Thermo Fisher Scientific). The labeled cells were seeded onto the coated plate (10^4^ cells per well) and cultured for 5 days in a 37 °C CO_2_ incubator (DAIHAN Scientific). The CellTrace Violet dilution was assessed with flow cytometry.

### scRNA-seq

The cells were resuspended in PBS and filtered through a 40 µm filter. After the cells were counted using LUNA-FL Automated Fluorescence Cell Counter (Logos Biosystems), 10x Genomics Chromium Instrument and complementary DNA synthesis kit (Chromium Next GEM Single Cell 5′ Kit v2 and Chromium Next GEM Chip K Single Cell Kit) were used to generate a barcoded cDNA library for single-cell RNA sequencing (scRNA-seq). The cDNA library quality was determined using an Agilent Bioanalyzer (Agilent technologies). Using this library, two paired-end 200 bp FlowCells were run on an Illumina NovaSeq6000 S2 Rgt Kit v1.5 (200 cycles (read lengths: 28 bp Read1, 10 bp I7 Index, 10 bp I5 Index, and 90 bp Read2) (Illumina). For T cell-receptor (TCR) sequencing, cDNA was used to process a nested-PCR enrichment method to increase specificity of the amplification of the constant region of the T cell transcripts. Using 10x Genomics Chromium Single Cell Human TCR Amplification Kit, the TCR libraries were enriched for alpha–beta TCRs. The TCR library quality was determined using an Agilent Bioanalyzer (Agilent technologies). Using this library, two paired-end 200 bp FlowCells were run on an Illumina NovaSeq6000 S2 Rgt Kit v1.5 (200 cycles (read lengths: 50 bp Read1, 10 bp I7 Index, 10 bp I5 index and 100 bp Read2) (Illumina). In practice, the demultiplexed filtered gene barcode matrices were loaded and merged. The merged matrix was processed with quality control, normalization and scaling. Cellular indexing of transcriptomes and epitopes (CITE) sequencing was merged as a new assay, and TCR information was merged as metadata for further analysis.

### Bioinformatics

All scRNA-seq analysis were carried out using Seurat R package. The clonotypes were defined using complementarity-determining regions (CDR) of TCRα and TCRβ chains acquired from scTCR sequencing. The number of unique clonotypes was assessed by counting duplicated clonotypes as one. Clonality and clonal diversity were calculated using Pielou’s evenness index (asbio R package) and Shannon diversity index (diverse R package), respectively. The virus-specific clonotypes were determined on the basis of the CDR3 sequences of the TCRα and TCRβ chains, which were considered virus specific if they both matched the same viral epitope-specific CDR3 sequences specified in VDJdb. A pseudotime analysis was carried out using monocle3 R package. The Seurat data were transformed into a monocle3-compatible format using SeuratWrappers R package. The transformed data were clustered using the Louvain method in a uniform manifold approximation and projection (UMAP). The *C0.Tn* cluster was selected as the beginning of the pseudotime. The calculated pseudotime was then merged as metadata to the former Seurat data and used for further analysis. The module scores were calculated in total CD8^+^ T cells using AddModuleScore function. The average module score of the CD27^+^CD28^+^*-Tem* cluster in each individual was calculated and used for further analysis. For some experiments, the following published scRNA-seq datasets from peripheral blood of healthy individuals and patients with viral infections or autoimmune diseases were used: COVID ATLAS (coronavirus disease 2019, COVID-19), GSE149689 (influenza virus), GSE202410 (human immunodeficiency virus, HIV), GSE182159 (hepatitis B virus, HBV), GSE156989 (systemic lupus erythematosus), GSE152316 (Crohn’s disease and spondyloarthritis), GSE138266 (multiple sclerosis), GSE125527 (ulcerative colitis), GSE185857 (palmoplantar pustulosis) and GSE157278 (primary Sjögren’s syndrome). The clusters with CD8^+^ CD27^+^CD28^+^-Tem signatures (*GZMH*^−^*GZMK*^*+*^) in these datasets were accessed and used for module score analysis. The relative module scores were calculated by subtracting each patient’s average module score with the average of that of healthy individuals from the same dataset.

### Protein expression analysis

To analyze protein expression changes between healthy individuals and patients, we accessed mean fluorescence intensity (MFI) of each protein using flow cytometry. To normalize for batch effects across experiments, a healthy individual was included in all experiments and served as the reference for analysis. For all proteins, the MFI of each subset was divided by the MFI of naive T cells of the reference healthy individual to get the relative MFI. For pseudotime analysis with proteins, the following molecules were analyzed using flow cytometry: CD8, CD3, CXCR4, CD45RA, CD95, CD27, CD28, CD73, CCR7 and CXCR3. The channel values of flow cytometric data were accessed and analyzed with Seurat R package. The Seurat data were scaled but not normalized and analyzed for UMAP. A pseudotime analysis was performed using monocle3 R package. Each CD8^+^ T cell subset was divided into one pseudotime interval, and the pseudotime distribution for each subset in healthy individuals and patients was accessed. For each pseudotime interval, the distribution of the patients’ subset was subtracted by the distribution of healthy individuals’ subset at that pseudotime to obtain the proportion difference. The average pseudotime was calculated for each individual in the indicated subsets and then used for comparison.

### Statistics

Statistical analysis was performed with Prism (GraphPad) or R. The statistical significances were tested with Mann–Whitney *U* test for unpaired samples or Wilcoxon matched-pairs for paired samples unless addressed otherwise. The statistical significance for biomarker performance was assessed using Fisher’s exact test for both best response and DCB. For the best response, only the statistical results for PR are presented. The significance for survival curves were tested using the Gehan–Breslow–Wilcoxon test. The linear regressions were calculated using an *F*-test for nonzero correlation. The values of ^∗∗∗∗^*P* < 0.0001, ^∗∗∗^*P* < 0.001, ^∗∗^*P* < 0.01 and ^∗^*P* < 0.05 were considered significant.

## Results

### Patients with lung cancer exhibit a stage-dependent progressive alteration in the composition of peripheral blood CD8^+^ T cell populations

**Fig. 1:**
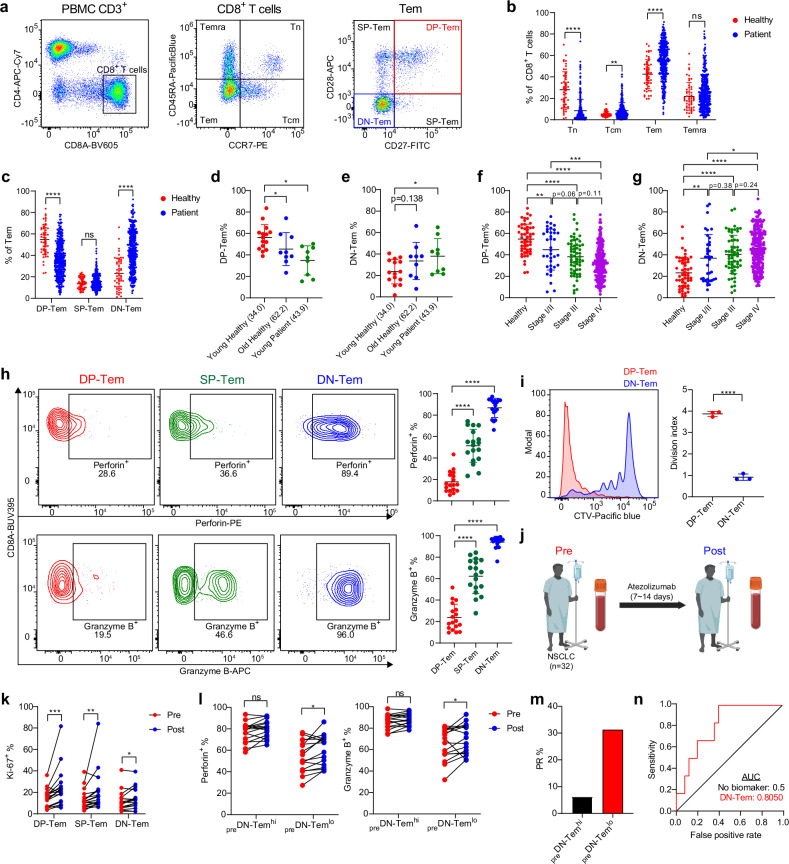
Patients with lung cancer show altered peripheral blood CD8^+^ Tem subsets. **a** The gating strategies for CD8^+^ T cell subsets. **b** CD8^+^ T cell subset frequencies in healthy individuals (*n* = 53) and patients with NSCLC (*n* = 349). **c** CD8^+^ Tem subset frequencies. **d**, **e** DP-Tem (**d**) and DN-Tem (**e**) frequencies in healthy individuals and patients of different ages. The numbers in brackets represent the mean age of the group. **f**, **g** DP-Tem (**f**) and DN-Tem (**g**) frequencies in patients at different stages of NSCLC. **h** Perforin^+^ and granzyme B^+^ frequencies in CD8^+^ Tem subsets. **i** CTV-dilution of DP-Tem and DN-Tem after 5 days of in vitro activation with plate-bound anti-CD3 (5 µg ml^−1^) and anti-CD28 (2 µg ml^−1^) antibodies. **j** An illustration of the blood collection process. **k** The changes in Ki-67^+^ cell frequency after ICI therapy in DP-Tem, SP-Tem and DN-Tem (*n* = 20). **l** The changes in perforin^+^ and granzyme B^+^ frequencies after ICI therapy in _pre_DN-Tem^hi^ (*n* = 15) and _pre_DN-Tem^lo^ (*n* = 15) groups. **m** The proportion of patients with PR to ICI therapy. **n** The ROC curve of DN-Tem and its AUC. All the bar graphs represent the mean ± standard deviation. ^∗∗∗∗^*P* < 0.001, ^∗∗∗^*P* < 0.001, ^∗∗^*P* < 0.01, ^∗^*P* < 0.05. ns, not significant; Tn, naive T cells; Tcm, central memory T cells; Tem, effector memory T cells; Temra, effector memory T cells reexpressing CD45RA; SP, single positive; CTV, CellTrace Violet.

To investigate whether tumors induce alterations in the composition of peripheral blood CD8^+^ T cells, we retrospectively analyzed CD8^+^ T cells in the PBMCs of 53 healthy donors and 349 patients with lung cancer (294 patients with NSCLC and 55 patients with SCLC) (Supplementary Table 1). Based on the expression of CCR7 and CD45RA (Fig. 1a), the CD8^+^ T cells were classified into naive (Tn; CCR7^+^CD45RA^+^), central memory (Tcm; CCR7^+^CD45RA^−^), effector memory (Tem; CCR7^−^CD45RA^−^) and effector memory reexpressing CD45RA (Temra; CCR7^−^CD45RA^+^). Interestingly, patients exhibited a higher frequency of Tem than healthy individuals (Fig. 1b). Moreover, when Tem were further subdivided by CD27 and CD28 expression (Fig. 1a, right), the patients showed a lower frequency of less-differentiated CD27^+^CD28^+^ double-positive (DP)-Tem and higher frequency of more-differentiated CD27^−^CD28^−^ double-negative (DN)-Tem compared with healthy individuals (Fig. 1c). While these changes were also observed in healthy individuals with age, they were particularly pronounced among patients even at younger ages (Fig. 1d, e) and consistently observed in age-matched comparisons (Supplementary Fig. 1a, b).

Notably, the decrease in DP-Tem frequency and increase in DN-Tem frequency observed in patients with NSCLC were associated with tumor progression (Fig. 1f, g) and were also evident in patients with extensive disease stage SCLC (Supplementary Fig. 1c). However, it is important to note that these changes were largely independent of age, sex, histologic classification, epidermal growth factor receptor mutation and, notably, tumor PD-L1 expression (Supplementary Fig. 1d–h). Further examination of functional properties revealed a substantial increase in the expression of cytotoxic molecules, perforin and granzyme B, during the transition from DP to DN subsets (Fig. 1h and Supplementary Fig. 1i). By contrast, consistent with the indispensable role of CD28 in proliferation and activation upon TCR stimulation in vitro (Supplementary Fig. 1j–m), substantially reduced proliferative responses were observed in DN-Tem relative to DP-Tem (Fig. 1i).

To further investigate whether these changes are related to clinical responses to ICI therapy, we retrospectively analyzed PBMCs isolated from the blood of 32 patients with NSCLC collected before (pre) and after (post) anti-PD-L1 therapy (Fig. 1j and Supplementary Table 2). A substantial increase in the Ki-67^+^ proliferating population was evident in post-therapy patients’ Tem (Supplementary Fig. 1n), but notably, this increase was prominent in DP-Tem but not DN-Tem (Fig. 1k). Likewise, an elevated frequency of post-therapy perforin^+^ and granzyme B^+^ Tem was only observed in patients with a relatively lower pretherapy DN-Tem frequency (_pre_DN-Tem^lo^; lower than median) but not those with a higher pretherapy DN-Tem frequency (_pre_DN-Tem^hi^; higher than median) (Fig. 1l). Importantly, _pre_DN-Tem^lo^ patients showed better therapeutic responses than _pre_DN-Tem^hi^ patients (33.3%, 95% confidence interval (CI) 9.48–57.19% versus 6.7%, 95% CI 1.19–29.82%; Fig. 1m and Supplementary Fig. 1o). The area under the curve (AUC) in the receiver operating characteristic (ROC) curve of DN-Tem was 0.8050 (95% CI 0.616–0.994; Fig. 1n). These data suggest a potential link between the altered composition in the CD8^+^ T cell compartment and therapeutic responses to ICI therapy.

### Increased CD8^+^ DN-Tem in patients with NSCLC result from generation of clonally diverse *GZMK*^*+*^*.DN-Tem*

**Fig. 2:**
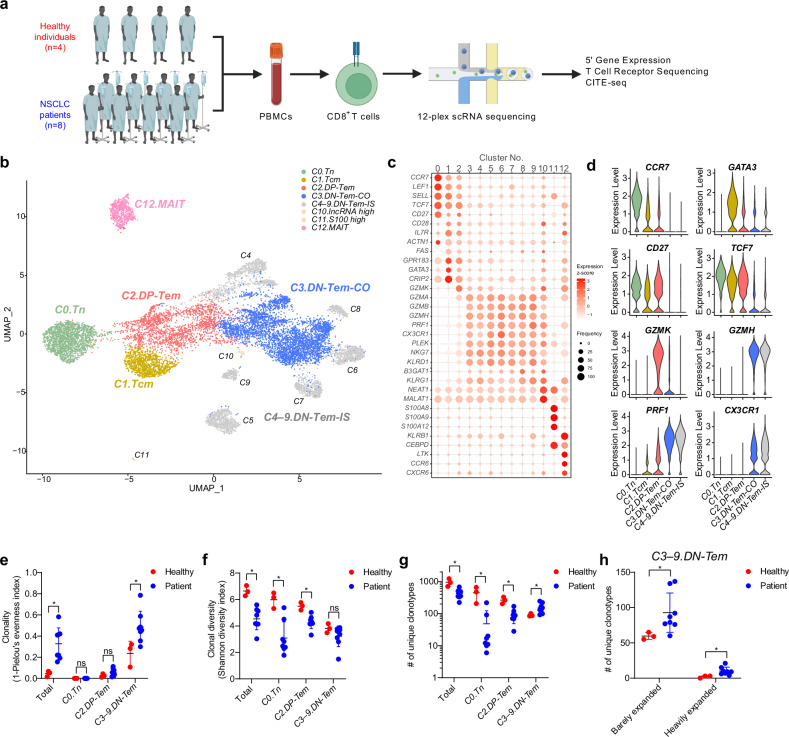
Increased CD8^+^ DN-Tem in patients with NSCLC rely on both clonal expansion-dependent and expansion-independent mechanisms. **a** The experimental scheme for scRNA-seq. **b** A UMAP of scRNA-seq data. The clusters were color coded according to their labels. **c** The expressions of signature genes in each cluster. The color and size represent relative average expression (*z*-score) and frequency, respectively. **d** The expression of key signature genes in the *C0–9* clusters. **e**–**g** Clonality (**e**), clonal diversity (**f**), and the number of unique clonotypes (**g**) of indicated clusters in healthy individuals (*n* = 3) and patients (*n* = 8). **h** The number of barely expanded clones and heavily expanded clones in *C3–9.DN-Tem*. All the bar graphs represent the mean ± standard deviation. ^∗∗^*P* < 0.01, ^∗^*P* < 0.05. ns, not significant; Tn, naive T cells; Tcm, central memory T cells; Tem, effector memory T cells; sc, single cell.

We investigated the mechanism underlying the increased frequency of DN-Tem in patients with NSCLC. For this, CD8^+^ T cells were isolated from PBMCs of four healthy individuals and eight patients with NSCLC (Supplementary Table 3). This was followed by scRNA-seq, scTCR sequencing and scCITE sequencing for analyzing gene expression profile, TCR repertoire and CD45RA protein expression, respectively (Fig. 2a). We classified 13 clusters (C0–C12) using an unsupervised clustering method (Fig. 2b). Among these, C0 and C1 clusters were designated as *C0.Tn* and *C1.Tcm*, based on high expression of Tn signature genes (*CCR7*, *LEF1*, *SELL*, *TCF7* and *ACTN1*) and Tcm signature genes (*GPR183*, *GATA3* and *CRIP2*), respectively, along with CD45RA protein expression (Fig. 2c, d and Supplementary Fig. 2a). The C2 cluster was defined as *C2.DP-Tem* based on its relatively high expression of early effector cell signature genes (*CD27*, *CD28* and *GZMK*) and low CD45RA protein expression (Fig. 2c, d and Supplementary Fig. 2a). Unlike the C2 cluster, the C3–C9 clusters exhibited high expression of late-effector cell signature genes (*GZMA*, *GZMB*, *GZMH*, *PRF1*, *CX3CR1*, *PLEK*, *NKG7*, *KLRD1*, *B3GAT1* and *KLRG1*) (Fig. 2c, d). These clusters showed somewhat heterogeneous CD45RA expression, with levels between those of Tem and Temra (Supplementary Fig. 2a). However, CD45RA^−^ and CD45RA^+^ cells within these clusters exhibited minimal transcriptomic differences (Supplementary Fig. 2b–g); therefore, for simplicity, they were collectively designated as *C3–9.DN-Tem*. Among the C3–C9 clusters, the C3 cluster was observed in all individuals analyzed, while the C4–C9 clusters were exclusively observed in specific individuals only and exhibited highly limited *TCRα* and *TCRβ* sequences as well as low TCR diversity (Supplementary Fig. 2h–j). Therefore, we designated them as ‘common’ *C3.DN-Tem* (*C3.DN-Tem-CO*) and ‘individual-specific’ *C4–9.DN-Tem* (*C4–9.DN-Tem-IS*), respectively. Moreover, the C10, C11 and C12 clusters showed high expression of long noncoding RNA genes (*NEAT1* and *MALAT1*), S100 genes (*S100A8*, *S100A9* and *S100A10*) and Mucosal-associated invariant T cell (MAIT) signature genes (*KLRB1*, *CEBPD*, *LTK*, *CXCR6* and *CCR6*), which were designated as *C10.lncRNA high*, *C11.S100 high* and *C12.MAIT*, respectively (Fig. 2c).

With reference to the C0–C9 clusters, patients with NSCLC had lower proportions of *C0.Tn* and *C2.DP-Tem* and higher proportions of *C3.DN-Tem-CO* and *C4–9.DN-Tem-IS* than healthy individuals (Supplementary Fig. 2h), consistent with the previous flow cytometry data (Fig. 1b, c). Among *C4–9.DN-Tem-IS*, the *C4* cluster showed exceptionally rare occurrence in only one (H2) of the four healthy individuals (Supplementary Fig. 2h); therefore, H2 was excluded from further analysis. Next, we performed TCR repertoire analysis to ascertain whether the elevated proportion of DN-Tem observed in patients with NSCLC involved oligoclonal expansion. As expected, the clonality of the mixture of clusters (C0–C9, representing total peripheral blood CD8^+^ T cells) markedly increased in patients compared with healthy individuals, while their diversity substantially decreased (Fig. 2e, f). However, we noticed that the reduced diversity was particularly prominent in the *C0.Tn* and *C2.DP-Tem* but notably was absent in the *C3–9.DN-Tem* (Fig. 2f), despite their increased clonality (Fig. 2e). Given that sample size plays a crucial role in diversity analysis, we explored whether the biased distribution of T cell subsets contributed to this phenomenon. Indeed, the uncoupling of clonality and diversity was due to the increase of number of unique clonotypes in *C3–9.DN-Tem* in patients relative to healthy individuals (Fig. 2g). Interestingly, the increase in the number of unique clonotypes in *C3–9.DN-Tem* was observed in heavily expanded clones (more than ten duplicates) and also to a greater extent in barely expanded clones (no duplicates) (Fig. 2h). Given that tumor-specific T cells in the circulation are typically found in expanded forms^38^, whereas the majority of T cells appear as singular clones^39^, such increase of barely expanded clones in *C3–9.DN-Tem* suggests an unexpected role of expansion-independent mechanism in increasing the size of *C3–9.DN-Tem* as well as maintaining diversity.

**Fig. 3:**
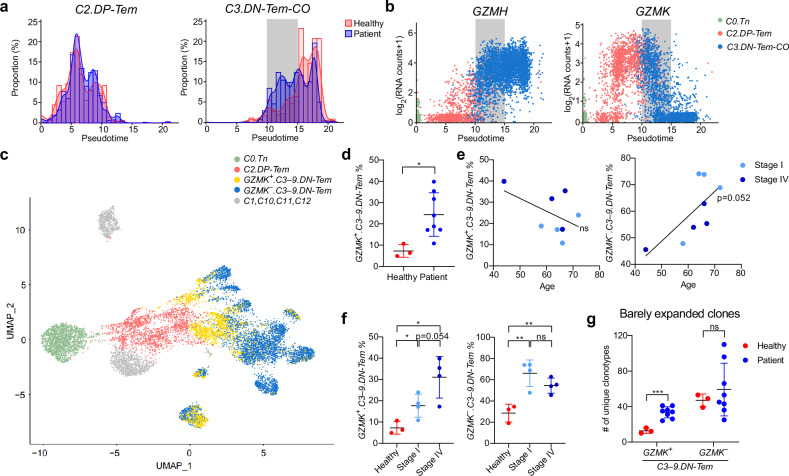
Increased CD8^+^ DN-Tem in patients with NSCLC are associated with clonally diverse *GZMK*^*+*^*.DN-Tem* generation. **a** A histogram for pseudotime of *C2.DP-Tem* and *C3.DN-Tem-CO*. The gray box indicates the pseudotime where patients and healthy individuals were different. **b** *GZMH* and *GZMK* expression by pseudotime. **c** A UMAP after *C3–9.DN-Tem* is divided into *GZMK*^*+*^*.C3–9.DN-Tem* and *GZMK*^*−*^*.C3–9.DN-Tem*. **d** *GZMK*^*+*^*.C3–9.DN-Tem* frequency in CD8^+^ T cells in healthy individuals (*n* = 3) and patients (*n* = 8). **e** The correlation between age and *GZMK*^*+*^*.C3–9.DN-Tem* or *GZMK*^*−*^*.C3–9.DN-Tem* frequency. The patients were divided into stage I and stage IV (*n* = 4 and 4, respectively). **f** *GZMK*^*+*^*.C3–9.DN-Tem* and *GZMK*^*−*^*.C3–9.DN-Tem* frequency in healthy individuals, patients with stage I NSCLC and patients with stage IV NSCLC (*n* = 3, 4 and 4, respectively). **g** The number of barely expanded clones in *GZMK*^*+*^*.C3–9.DN-Tem* and *GZMK*^*−*^*.C3–9.DN-Tem*. All the bar graphs represent the mean ± standard deviation. ^∗∗∗^*P* < 0.001, ^∗^*P* < 0.05. **e** The lines represent the linear regression. ns, not significant; Tn, naive T cells; Tem, effector memory T cells.

We investigated how the above unexpected clonal expansion-independent increase in DN-Tem arises. For this, we further characterized the *C3.DN-Tem-CO* based on pseudotime and compared any potential differences between patients and healthy individuals (Supplementary Fig. 3a). Interestingly, a marked difference was apparent especially at relatively earlier pseudotime points for *C3.DN-Tem-CO*, as evidenced by a substantially higher fraction of cells in patients than in healthy individuals (Fig. 3a, gray box). These cells observed in the *C3.DN-Tem-CO* expressed *GZMH* at high levels and interestingly also expressed *GZMK* (Fig. 3b). Therefore, *C3–9.DN-Tem* were further characterized into two clusters: *GZMK*^*+*^*.C3–9.DN-Tem* and *GZMK*^*−*^*.C3–9.DN-Tem* (Fig. 3c). *GZMK*^*+*^*.C3–9.DN-Tem* were unusual cell types expressing both early and late-effector genes (Supplementary Fig. 3b). Flow cytometric analysis confirmed that granzyme K^+^ DN-Tem represent a less differentiated form of DN-Tem, maintaining intermediate features while remaining distinct from DP-Tem (Supplementary Fig. 3c–n).

Notably, *GZMK*^*+*^*.C3–9.DN-Tem* was nearly absent in healthy individuals but markedly elevated in patients (Fig. 3d and Supplementary Fig. 3o). Moreover, while the frequency of *GZMK*^*−*^*.C3–9.DN-Tem* was associated with age, the frequency of *GZMK*^*+*^*.C3–9.DN-Tem* was dependent on tumor stage (Fig. 3e, f). Furthermore, the increased number of barely expanded clones observed in *C3–9.DN-Tem* (Fig. 2h) was primarily attributable to *GZMK*^*+*^*.C3–9.DN-Tem* rather than *GZMK*^*−*^*.C3–9.DN-Tem* (Fig. 3g). All these observations support the notion that the increased DN-Tem frequency observed in peripheral blood CD8^+^ Tem in NSCLC patients was primarily due to increase of clonally diverse *GZMK*^*+*^*.DN-Tem* population.

### Peripheral blood CD8^+^ T cells in patients with NSCLC exhibit altered transcriptional signatures associated with reduced T cell quiescence

**Fig. 4:**
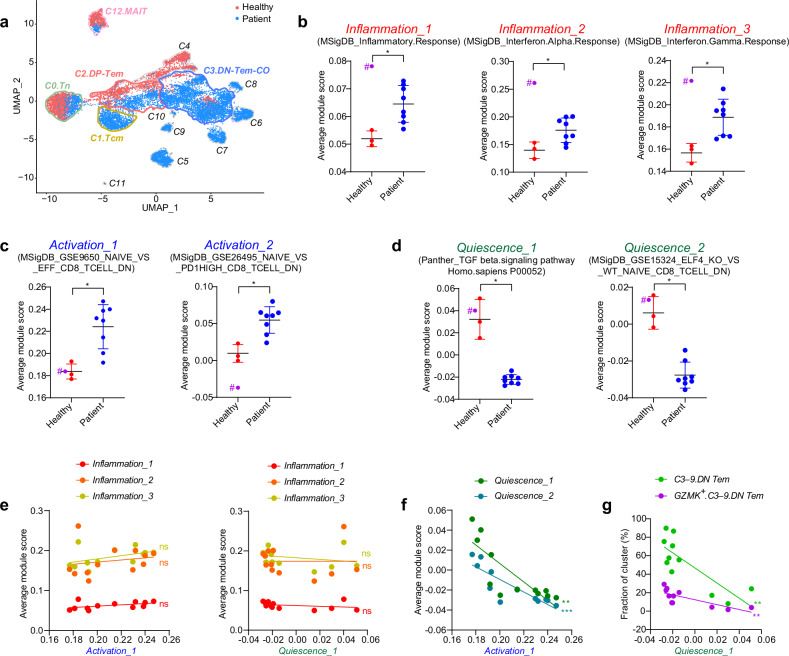
Peripheral blood CD8^+^ T cells in patients with NSCLC exhibit altered transcriptional signatures indicative of loss of quiescence. **a** A UMAP color coded according to disease status. The colored lines represent boundaries for each cluster. **b**–**d** The average module scores of gene sets related to inflammation (**b**) T cell activation (**c**) and T cell quiescence (**d**) in *C2.DP-Tem* of healthy individuals (*n* = 4) or patients (*n* = 8). The purple dots represent one of the healthy individual (H2). The samples with # were excluded from statistics. **e** Correlations between inflammation gene sets and T cell activation or T cell quiescence gene sets (*n* = 12). **f** The correlation between T cell activation and T cell quiescence gene sets. **g** The correlation between a T cell quiescence gene set and frequencies of *C3–9.DN-Tem* or *GZMK*^*+*^*.C3–9.DN-Tem*. All the bar graphs represent the mean ± standard deviation. ^∗∗∗^*P* < 0.001, ^∗∗^*P* < 0.01, ^∗^*P* < 0.05. **e**–**g** The lines represent the linear regression. The significance represents nonzero correlation. Tn, naive T cells; Tcm, central memory T cells; Tem, effector memory T cells.

The increased frequency of clonally diverse *GZMK*^*+*^*.DN-Tem* in patients with NSCLC raises the question of whether this increase is solely due to tumor antigen-specific responses. In fact, when compared with the 75,302 known virus-specific CDR3 sequences in VDJdb^40^, a fraction of *GZMK*^*+*^*.DN-Tem* (particularly *GZMK*^*+*^*.C3.DN-Tem-CO*) contained clonotypes restricted to known viral epitopes (Supplementary Fig. 4a), suggesting that not all *GZMK*^*+*^*.DN-Tem* are tumor specific. We therefore further investigated the mechanism of how tumor nonspecific *GZMK*^*+*^*.DN-Tem* could be increased in patients with NSCLC. Interestingly, substantial differences in gene expression profiles were observed in *C2.DP-Tem*, *C0.Tn*, *C3.DN-Tem* and even *C12.MAIT*, as evidenced by clearly distinguishable positions in the UMAP (Fig. 4a). This suggests that tumor-associated transcriptomic changes occur broadly and systemically across various cell types in peripheral blood.

Next, to investigate how the transcriptomic changes are associated with the generation of *GZMK*^*+*^*.C3–9.DN-Tem*, we conducted a pathway analysis in its adjacent precursor, *C2.DP-Tem*. For this, we selected 644 signal and immune-related gene sets from public databases (Broad Institute, PANTHER) (Supplementary Information 2), which were categorized into four clusters, namely gene set clusters (GSC) 1, 2, 3 and 4, according to the module scores of *C2.DP-Tem* (Supplementary Fig. 4b). GSC1 and GSC2 showed notable overall increases in patients relative to the levels in healthy individuals, while GSC3 was at a similar level in both groups (Supplementary Fig. 4b). By contrast, GSC4 was markedly increased in healthy individuals relative to the levels in patients (Supplementary Fig. 4b). Notably, in the healthy group, one individual (H2), who was excluded from the previous analysis (Figs. 2 and 3), was observed to have a high GSC1 expression level, even higher than all the patients (Supplementary Fig. 4b), which led to placing H2 in a distinctly different position from the other healthy individuals in the principal component analysis (Supplementary Fig. 4c).

Next, we analyzed the key functional gene sets within each GSC (Fig. 4b–d). GSC1 and GSC2, which were notably higher in patients than in healthy individuals, included gene sets associated with inflammation (Fig. 4b) and T cell activation (Fig. 4c), respectively. By contrast, GSC4 contained gene sets related to T cell quiescence^41–45^, and these were expressed at lower levels in patients than in healthy individuals (Fig. 4d). Interestingly, H2 in healthy individuals showed high inflammation (GSC1); however, activation (GSC2) and quiescence (GSC4) remained unchanged (Fig. 4b–d, purple). Importantly, in the examination of the expression correlations between these gene sets, inflammation did not correlate at all with activation or quiescence (Fig. 4e), whereas quiescence and activation were strongly inversely correlated (Fig. 4f). Moreover, the decreased quiescence was closely associated with the increase of *C3–9.DN-Tem* and *GZMK*^*+*^*.C3–9.DN-Tem* (Fig. 4g).

To determine whether the above phenomenon is cancer-specific or common to diseases involving infection or inflammation, we analyzed published scRNA-seq data from patients with various viral infections and autoimmune diseases (COVID Atlas, Gene Expression Omnibus). The patients with COVID-19 indeed exhibited higher inflammation, as expected, than healthy individuals (Supplementary Fig. 4d). However, activation and quiescence remained similar to that of healthy individuals (Supplementary Fig. 4e, f). Remarkably, similar to patients with COVID-19, quiescence was not notably different from that of healthy individuals among patients with other acute and even chronic viral infections (Supplementary Fig. 4g, yellow box). However, interestingly, various autoimmune diseases showed varying degrees of differences in quiescence (Supplementary Fig. 4g, green box). Moreover, the decrease in quiescence was not associated with inflammation but was strongly linked to the increase in the activation (Supplementary Fig. 4h). Furthermore, the decrease in quiescence showed strong correlation with increase in *GZMK*^*+*^*GZMH*^*+*^ population (Supplementary Fig. 4i). This phenomenon, which was common with several autoimmune diseases and lung cancer, strongly supports the notion that unrestrained T cell quiescence is closely associated with homeostatic dysregulation, resulting in increased T cell activation and unregulated differentiation.

### Peripheral blood CD8^+^ T cells in patients with NSCLC exhibit altered phenotypic signatures indicative of a continuous flow of T cell activation and differentiation

**Fig. 5:**
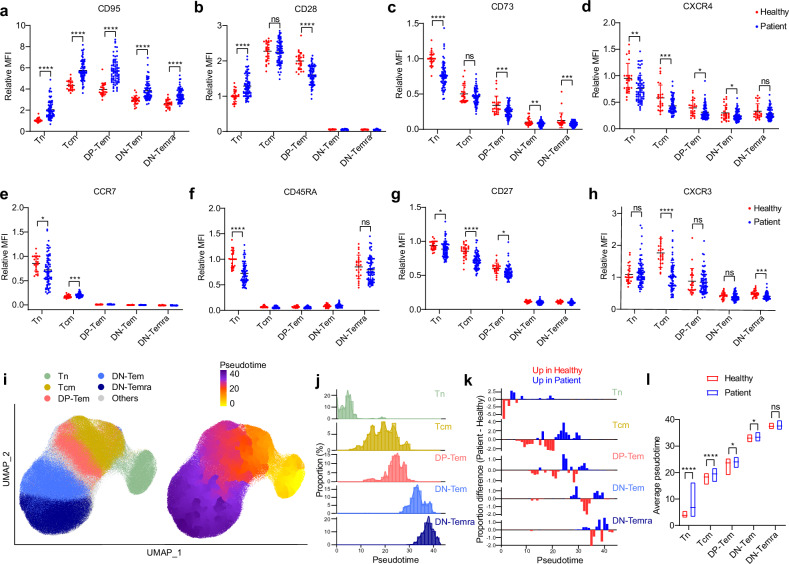
Peripheral blood CD8^+^ T cells in patients with NSCLC exhibit gross protein expression alterations. **a**–**h** The relative MFI of CD95 (**a**), CD28 (**b**), CD73 (**c**), CXCR4 (**d**), CCR7 (**e**), CD45RA (**f**), CD27 (**g**), and CXCR3 (**h**) in each subset from healthy individuals (*n* = 25) and patients with stage IV NSCLC (*n* = 71). The relative MFI was calculated by dividing MFIs with that of Tn from a healthy individual. The bar graphs represent the mean ± standard deviation. **i** A UMAP generated with protein expressions. The cells were color coded according to their labels (left) or by pseudotime (right). **j** A histogram of pseudotime in each subset. **k** The proportion difference between patient and healthy individuals in each pseudotime point. The red indicates pseudotime where healthy individuals were more abundant than patients, while blue indicates the opposite. **l** The average pseudotime of each subset in healthy individuals and patients. The lines at the floating bars represent mean with error bars (minimum to maximum). ^∗∗∗∗^*P* < 0.0001, ^∗∗∗^*P* < 0.001, ^∗∗^*P* < 0.01, ^∗^*P* < 0.05. Tn, naive T cells; Tcm, central memory T cells; Tem, effector memory T cells; Temra, effector memory T cells reexpressing CD45RA.

Based on the above gene expression alterations indicative of decreased quiescence, we investigated whether peripheral blood CD8^+^ T cells from patients with NSCLC show phenotypic signatures following such genetic alterations. Notably, there were substantial differences in the expression levels of various markers related to activation and/or differentiation in peripheral CD8^+^ T cell subsets (CD95, CD28, CD73, CXCR4, CCR7, CD45RA, CD27 and CXCR3) (Fig. 5a–h and Supplementary Fig. 5a). In particular, CD95 expression, known to increase upon TCR stimulation, was substantially higher in patients with NSCLC than in healthy individuals, and surprisingly, this difference was observed across the entire CD8^+^ T cell compartment comprising Tcm, DP- and DN-Tem, DN-Temra and even Tn (Fig. 5a). Similar phenomena were also observed in patients with SCLC (Supplementary Fig. 5b–i). When the CD8^+^ T cell compartment was further subclustered by pseudotime analysis on the basis of the above protein expression changes (Fig. 5i, j), nearly all subsets (especially in Tn, Tcm and DP-Tem) in patients showed enhanced occurrence at the later pseudotimes relative to healthy individuals (Fig. 5k, l). These results further support the notion that there is an alteration in both gene and even protein expression across all peripheral blood CD8^+^ T cell populations in patients with lung cancer, leading to a continuous flow of cell differentiation.

### Cancer-associated systemic alterations in peripheral blood CD8^+^ T cell homeostasis become predictors of clinical outcomes after ICI therapy

**Fig. 6:**
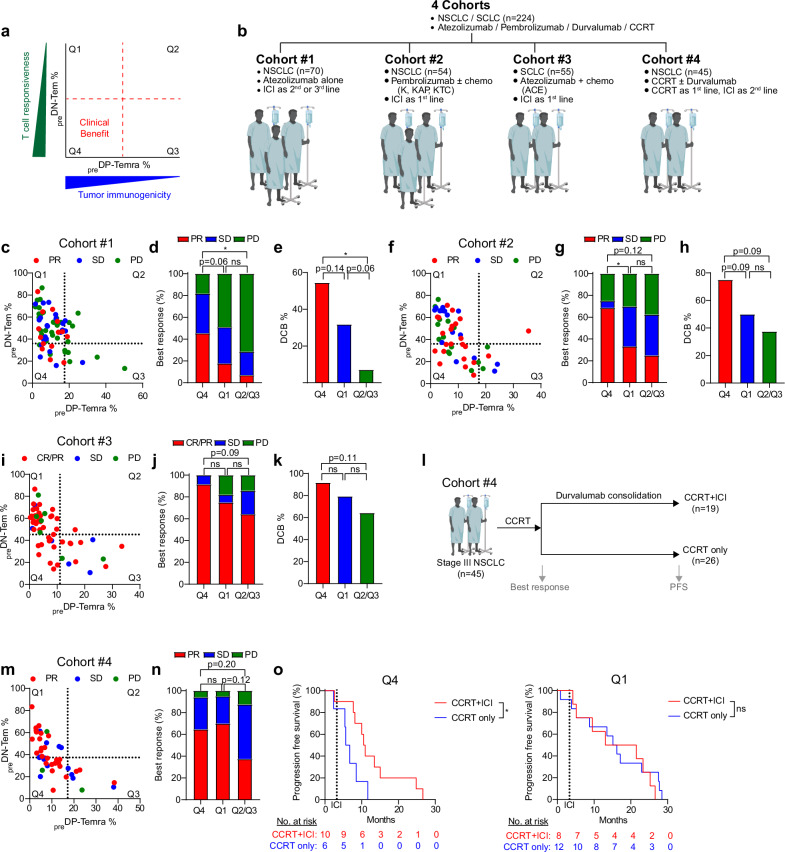
Cancer-associated homeostatic dysregulation in systemic CD8^+^ T cells correlates with ICI responsiveness. **a** A strategy to predict clinical benefit using two CD8^+^ T cell subsets. **b** A summary of cohorts. **c**–**k** The relationship between frequencies of _pre_DN-Tem and _pre_DP-Temra and clinical responses in cohort 1 (*n* = 70) (**c**–**e**) cohort 2 (*n* = 54) (**f**–**h**) and cohort 3 (*n* = 55) (**i**–**k**). In **c** **f** and **i** the *XY* graphs are generated with _pre_DN-Tem and _pre_DP-Temra frequencies from pretherapy peripheral CD8^+^ T cells. Each dot represents a patient. The colors represent best response at 3 months post-therapy. The dotted lines represent the threshold used to determine the four quadrants (Q1, Q2, Q3 and Q4) in the graph. In **d** **g** and **j**, the best response at 3 months post-therapy in patients in different quadrants is shown. In **e**, **h** and **k**, the proportion of patients with DCB at 6 months post-therapy in patients in different quadrants is shown. **l** The treatment strategy for cohort 4. All the patients were treated with CCRT as a first-line therapy. After 3 months, the best response for CCRT treatment was assessed. Subsequently, some patients concluded treatment with CCRT alone (CCRT only), while others underwent further treatment with durvalumab consolidation (CCRT + ICI). The PFS following durvalumab consolidation was then evaluated. **m**–**n** The relationship between frequencies of _pre_DN-Tem and _pre_DP-Temra and best responses to CCRT. **o** A comparison of the PFS between patients with different treatment strategies (CCRT only versus CCRT + ICI) within the patients in Q4 or Q1. The dotted lines represent the starting period of ICI. The statistical significance for biomarker performance was assessed using a Fisher’s exact test for PR or DCB. ^∗^*P* < 0.05. ns, nonsignificant; SCLC, small cell lung cancer; K, pembrolizumab monotherapy; KAP, pembrolizumab, pemetrexed and cisplatin combination therapy; KTC, pembrolizumab, paclitaxel and carboplatin combination therapy; ACE, atezolizumab, carboplatin and etoposide combination therapy; CR, complete response; SD, stable disease; PD, progressive disease; PFS, progression free survival; Tem, effector memory T cells; Temra, effector memory T cells reexpressing CD45RA.

Given the observed gene and protein expression alterations indicative of tumor-associated homeostatic dysregulation, we investigated the potential relationships between these phenomena and the clinical response to ICI therapy. In this regard, we previously reported a strong inverse correlation between CD8^+^ DP-Temra frequency in peripheral blood of patients with NSCLC and the abundance of tumor-infiltrating CD8^+^ T cells as well as high tumor immunogenicity, with predictive potential for ICI therapy responses^26^. We therefore hypothesized that a state of low pretreatment DP-Temra frequency (_pre_DP-Temra^lo^: indicative of high tumor immunogenicity) and low DN-Tem frequency (_pre_DN-Tem^lo^: indicative of high T cell responsiveness) in patients with NSCLC would induce the best ICI treatment responses (Fig. 6a). To address this possibility, we retrospectively analyzed _pre_DN-Tem and _pre_DP-Temra frequencies in the peripheral blood of 224 patients with lung cancer across four independent cohorts (Fig. 6b).

In our initial analysis of a cohort of 70 patients with NSCLC who received anti-PD-L1 (atezolizumab) monotherapy as a second or third line (cohort 1; Supplementary Table 4), PR was rarely observed in patients with _pre_DP-Temra^hi^ (>17.2%, Q2 and Q3), whereas it was clearly evident in patients with _pre_DP-Temra^lo^ (<17.2%, Q1 and Q4) (Fig. 6c, d). Particularly, among patients with _pre_DP-Temra^lo^ (Q1 and Q4), those with _pre_DN-Tem^lo^ (<36%, Q4) exhibited higher PR (45.5%, 95% CI 16.3–74.9% versus 17.8%, 95% CI 6.6–29.0%) and DCB (54.5%, 95% CI 25.1–84.0% versus 31.8, 95% CI 18.1–45.6%) post-ICI therapy than those with _pre_DN-Tem^hi^ (>36%, Q1) (Fig. 6d, e). Moreover, this association with clinical responses was also observed in patients receiving first-line anti-PD-1 (pembrolizumab) therapy in combination with chemotherapy (cohort 2; Supplementary Table 5) with higher PR (68.8%, 95% CI 46.0–91.5% versus 33.3%, 95% CI 16.5–50.2%) and DCB (75%, 95% CI 53.8–96.2% versus 50%, 95% CI 32.1–67.9%) in Q4 patients compared with Q1 (Fig. 6f–h). Similar trends were again observed in patients with SCLC who received first line combined anti-PD-L1 (atezolizumab) and chemotherapy (cohort 3; Supplementary Table 6). Although this combination therapy resulted in overall high response rate, Q4 patients tended to show better clinical response compared with Q1 patients in both PR (91.7%, 95% CI 76.0–107.3% versus 75.0%, 95% CI 59.0–91.0%) and DCB (91.7%, 95% CI 76.0–107.3% versus 79.3%, 95% CI 64.6–94.1%) (Fig. 6i–k). Although statistical analysis between Q4 and Q1 groups within individual cohorts was limited by small sample sizes, combining data from all three cohorts yielded statistically significant differences between Q4 and Q1 for both PR and DCB (Supplementary Fig. 6a). Furthermore, considering the age-associated increase in *GZMK*^−^ DN-Tem cells (Fig. 3e), limiting the prognosis analysis to relatively younger patients—thereby enhancing the detection of *GZMK*^+^ DN-Tem within the total DN-Tem population—resulted in a more pronounced distinction in biomarker performance between the Q4 and Q1 groups (Supplementary Fig. 6b, c).

By contrast, patients who received CCRT (cohort 4), half of whom received anti-PD-L1 (durvalumab) consolidation afterwards (Fig. 6l and Supplementary Table 7), showed no difference in the initial clinical outcomes to CCRT between Q4 and Q1 (Fig. 6m, n); however, the clinical benefit of anti-PD-L1 consolidation was observed exclusively in Q4 but not Q1 (Fig. 6o). These data from patients with lung cancer strongly support an immunological link between these two events, namely, altered DN-Tem (and DP-Temra) frequency and therapeutic responses to ICI.

We next investigated whether the predicted difference in ICI responses based on _pre_DN-Tem/_pre_DP-Temra was associated with tumor PD-L1 expression widely used in clinics. However, the higher ICI response rates of Q4 patients were not associated with tumor PD-L1 expression (Supplementary Fig. 6d), and indeed, within cohort 1, tumor PD-L1 expression did not affect ICI therapy responses (Supplementary Fig. 6e, f). Furthermore, when compared with various clinicopathologic variables, no clinical information other than _pre_DN-Tem was significantly associated with ICI therapy responses (Supplementary Fig. 6g). Collectively, these results suggest that the cancer-associated systemic alterations in peripheral blood CD8^+^ T cell populations can substantially influence clinical responses to ICI therapy.

## Discussion

The immunity cycle between tumors and antigen-specific CD8^+^ T cells is a major barrier against the malignant transformation of cancer^46,47^. However, tumors also adapt various resistance mechanisms against CD8^+^ T cells, including engagement of immune checkpoints^33,34,48,49^. Although a state of exhaustion is known to inhibit tumor-reactive CD8^+^ T cells within tumor tissues^50–52^, how tumors disrupt systemic CD8^+^ T cell populations to promote malignancy is less understood. In this study with patients with lung cancer, we demonstrated homeostatic dysregulation of peripheral blood CD8^+^ T cells with loss of quiescence and increased spontaneous activation/differentiation. This process occurred systemically and progressively during tumor progression, leading to gradual accumulation of DN-Tem that are refractory to TCR-driven proliferation and, moreover, strongly correlated with poor clinical responses to ICI therapy. Therefore, these findings suggest a new cancer evasion mechanism that could potentially avoid tumor-reactive CD8^+^ T cells at the systemic level.

The most notable feature seen in our patient cohort was the remarkable increase in the DN-Tem frequency. Given the high expression of cytotoxic molecules in DN-Tem, we assumed that tumor-associated, chronic antigenic stimulation would primarily contribute to this phenomenon. However, since most, if not all, DN-Tem are likely to be tumor nonspecific bystander cells^30,31,53–55^, we also postulated a process involving antigen-independent DN-Tem accumulation. Indeed, our scRNA-seq data provided strong evidence that these two possibilities occur simultaneously. As expected, increased clonality in DN-Tem was evident in patients, clearly supporting a role for oligoclonal expansion. However, the most surprising and unexpected data was that despite the increase in clonality, TCR diversity of DN-Tem did not decrease due to an unexpected increase in the number of unique clonotypes with little expansion that had an unusual phenotype of *GZMK*^*+*^ DN-Tem. Therefore, the accelerated accumulation of DN-Tem in patients is thought to occur, at least in part, through a continuous proliferation-independent differentiation from DP-Tem to *GZMK*^*+*^ DN-Tem during tumor progression.

The fact that *GZMK*^*+*^ DN-Tem abundance was greater in patients than in healthy individuals and had tumor nonspecific bystander (viral-specific) clones raised an intriguing question as to its generation mechanisms. Initially, we thought that cancer-associated chronic inflammation, which is common in malignant diseases^56–59^, could be a key factor in accelerating the DP-to-DN transition and promoting the generation of *GZMK*^*+*^ DN-Tem. In fact, our pathway analysis revealed markedly increased inflammation signatures in patients with lung cancer. However, patients with lung cancer as well as various viral infections and even autoimmune diseases showed no correlation between inflammation and T cell activation signatures, suggesting that different mechanisms other than cancer-associated chronic inflammation might be involved. Unlike the inflammation signatures, peripheral blood CD8^+^ T cells from patients with lung cancer showed markedly reduced quiescence signatures with a strong inverse correlation with activation signatures. We also confirmed that such low quiescence and high activation signatures were closely linked to an increase in DN-Tem (and *GZMK*^*+*^ DN-Tem*)*. These findings suggest that alterations in the ability to maintain systemic CD8^+^ T cells in a quiescent state during tumor progression promote uncontrolled activation/differentiation of systemic CD8^+^ T cell populations.

Since DN-Tem from patients showed high levels of cytotoxicity, we assume that if there are tumor-reactive cells within this subset, they are fully functional to exert potent cytotoxic activity. However, we showed that DN-Tem was almost incapable of proliferating upon TCR stimulation in vitro and was also poorly responsive to proliferate after ICI therapy. Therefore, rather than disrupting the cytotoxic function of DN-Tem per se, cancer is thought to induce a persistent homeostatic perturbation that switch cells from high division potentials (DP-Tem) to low division potentials (DN-Tem) in response to antigenic stimulation. This notion is reinforced by our intensive retrospective analysis data from four independent cohorts of patients with lung cancer receiving anti-PD-(L)1 therapy, which showed that _pre_DN-Tem^hi^ patients exhibited poorer clinical responses compared with _pre_DN-Tem^lo^ patients.

Building on the above relationship between _pre_DN-Tem frequency and post-ICI response, we propose a new mechanism by which cancer evades attack of potentially tumor-reactive CD8^+^ T cells. As such, cancer induces homeostatic dysregulation with loss of T cell quiescence and uncontrolled activation/differentiation across the entire CD8^+^ T cell populations. This is followed by a progressive accumulation of DN-Tem. Despite its high cytotoxic activity, DN-Tem has a low proliferative potential upon antigenic TCR stimulation, therefore causing a gradual shift in the fitness of potentially tumor-specific CD8^+^ T cells to a nonreactive state. We now named this phenomenon ‘Cancer-associated homeostatic dysregulation accelerating uncontrolled differentiation of systemic CD8^+^ T cells (CHAOS)’ (Supplementary Fig. 6h). The CHAOS model underscores the critical role of time in diminishing the ability of systemic CD8^+^ T cells to respond to ICI therapy. Over time, this dysregulation may prevent the clonal replacement of T cells in the TME from the periphery, ultimately leading to a failure in clinical response to ICI therapy (Supplementary Fig. 6h). Future studies are needed to validate whether CHAOS correlates with impaired clonal replacement following ICI therapy.

Under the condition of CHAOS, we suggest that even patients with cancer who could have responded to ICI therapy will eventually accumulate DN-Tem and become nonresponders. Therefore, it will be important to explore the possibility that patients with stage IV cancer who are _pre_DN-Tem^hi^—presumably ICI nonresponders based on CHAOS—respond much better to alternative therapies other than ICI. As a result, we uncover a new mechanism of cancer immune evasion that has important clinical implications for improving our understanding of the interactions between cancer and CD8^+^ T cells and for developing effective therapeutic strategies for patients with cancer.

## Supplementary Information

Supplementary Information

Supplementary Information 2

## Data Availability

All data needed to evaluate the conclusions in the study are present here and/or in the Supplementary Materials. scRNA-seq data have been deposited at the GEO (GSE247754) and are publicly available as of the date of publication.
