# Supplementary material for: Progressive accumulation of circulating CD27−CD28− effector/memory CD8+ T cells in patients with lung cancer blunts responses to immune checkpoint inhibitor therapy
Source: Exp Mol Med. 2025 May 1;57(5):1005–16. doi: 10.1038/s12276-025-01448-7 (PMC12130471; doi:10.1038/s12276-025-01448-7)
Supplement: Supplementary file 1 — Supplementary Information [file 12276_2025_1448_MOESM1_ESM.pdf]

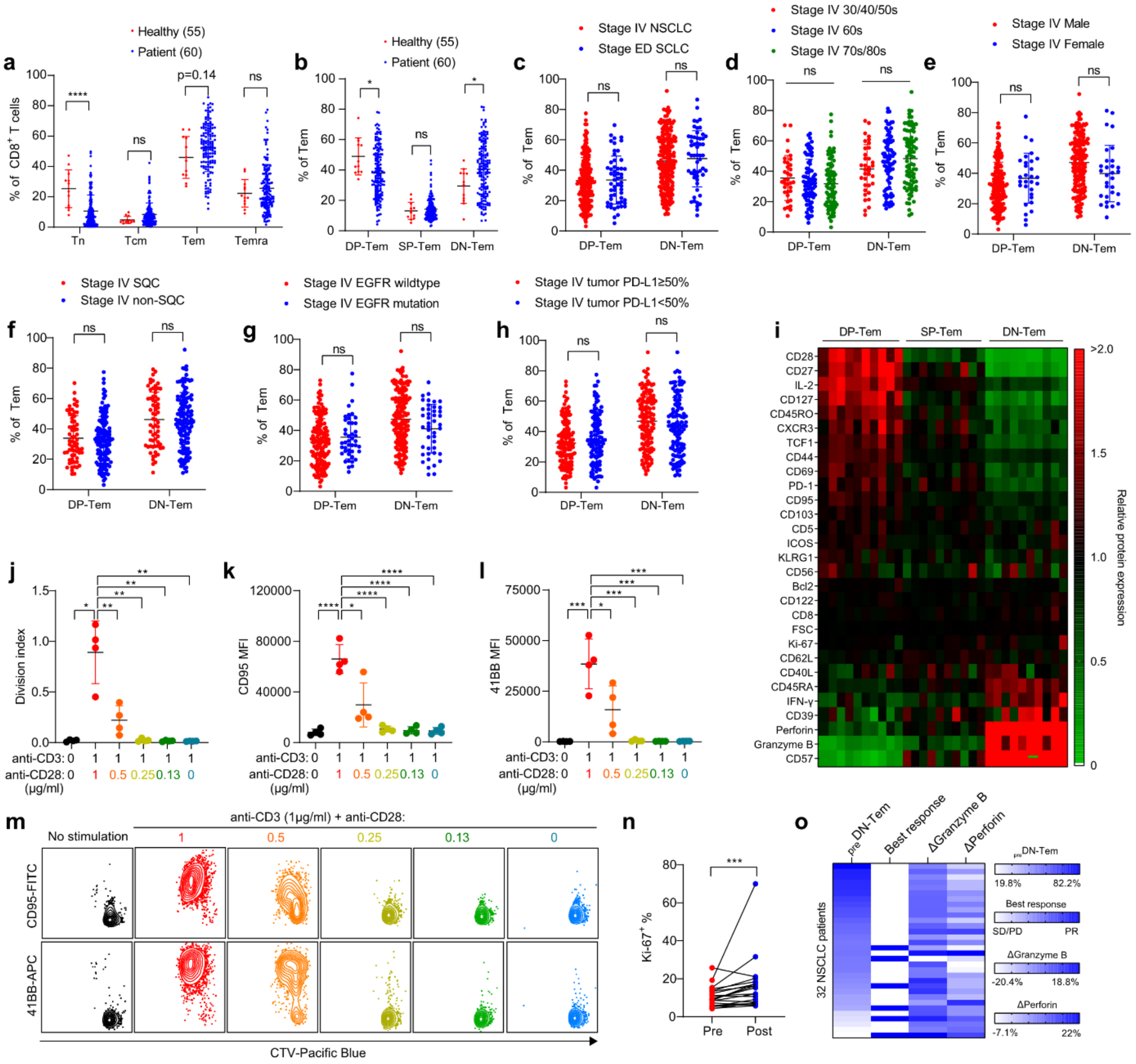

Supplementary Fig. 1. Lung cancer patients exhibit stage-dependent accumulation of a more-differentiated subset of peripheral blood CD8⁺ Tem.

(a–b) Age-matched comparisons for (a) CD8⁺ T cell subset frequencies and (b) CD8⁺ Tem subset frequencies in healthy individuals (n=12) and patients (n=144). Individuals with age between 40 to 70 were selected. Numbers in brackets represent mean age of the group.

(c) DP-Tem and DN-Tem frequencies in stage IV NSCLC patients or in stage ED SCLC patients (n=197 and 55, respectively).

(d–h) DP-Tem and DN-Tem frequencies in stage IV NSCLC patients grouped by (d) age (n=35, 80, and 82), (e) sex (n=167 and 30), (f) histological classification (n=65 and 132), (g) EGFR mutation (n=153 and 44), or (h) tumor PD-L1 (n=133 and 111).

(i) Relative protein expressions of various molecules in Tem subsets (n=10). Relative expressions were calculated by dividing MFIs with that of total Tem. Expressions were color-coded from green (low) to red (high).

(j-m) CTV-labeled naïve CD8 T cells were purified from PBMCs and cultured with indicated plate-bound antibodies. After 3 days, cells were analyzed for (j) division and two activation markers, (k) CD95 and (l) 41BB. (m) Representative flow cytometry plots are shown.
(n) Changes in Ki-67$^+$ cell frequency after ICI therapy in Tem (n=20).
(o) Heatmap illustrating the pre-therapy DN-Tem % ($_{pre}$DN-Tem), ICI therapy response (Best response), △Granzyme B (defined as $_{post}$Granzyme B% – $_{pre}$Granzyme B% in Tem), and △Perforin ($_{post}$Perforin% – $_{pre}$Perforin% in Tem) in 32 NSCLC patients. Values are color-coded from white to blue, as indicated in the legends.
All bar graphs represent mean ± SD, ****p<0.0001, ***p<0.001, *p<0.05. ns, non-significant; Tn, naïve T cells; Tcm, central memory T cells; Tem, effector memory T cells; Temra, effector memory T cells re-expressing CD45RA; DP, double-positive; DN, double-negative; NSCLC, non-small cell lung cancer; SCLC, small cell lung cancer; ED, extensive disesase; SQC, squamous cell carcinoma; EGFR, epidermal growth factor receptor; IL, interleukin; PD, programmed cell death; MFI, mean fluorescence intensity; ICI, immune checkpoint inhibitor.

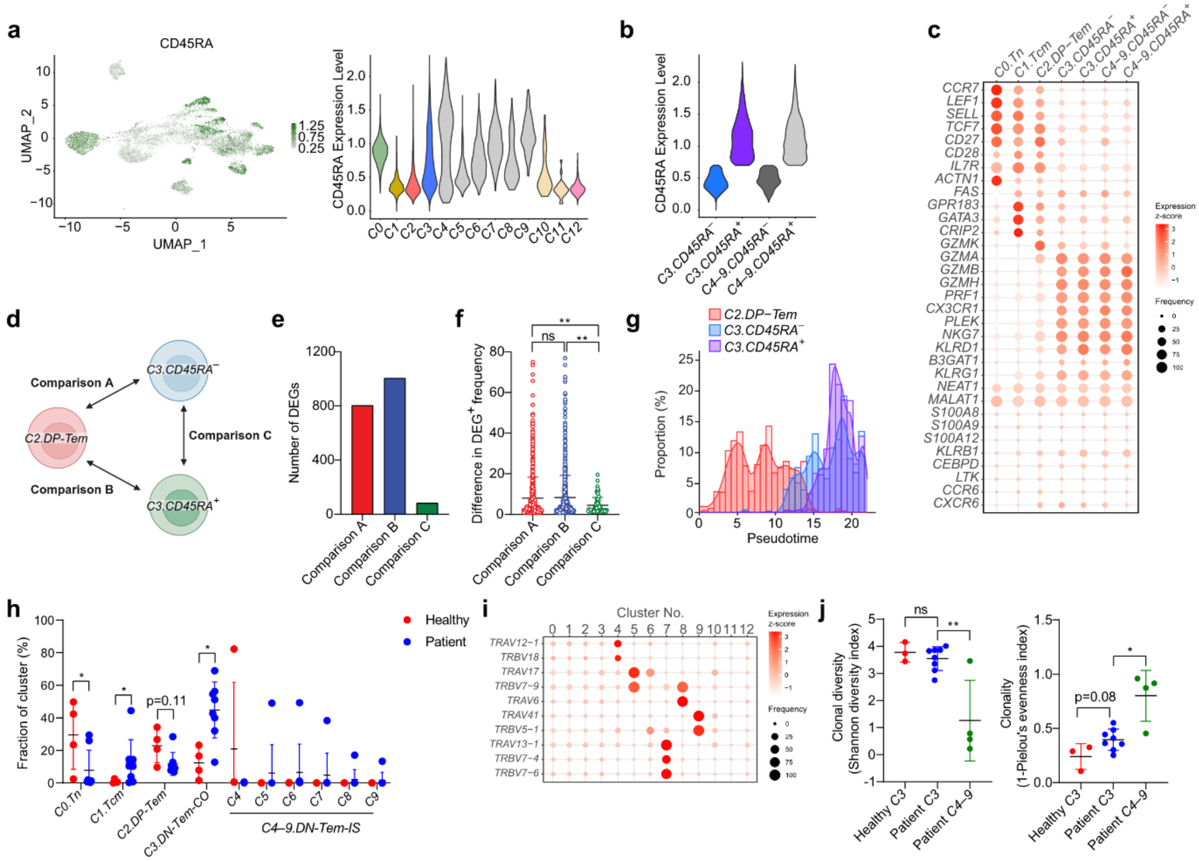

Supplementary Fig. 2. CD8⁺ T cell clusters in NSCLC patients.

(a) UMAP and violin plot of CD45RA expression measured using CITE-seq.

(b) The *C3.DN-Tem-CO* and *C4–C9.DN-Tem-IS* clusters were further subclustered into CD45RA⁻ and CD45RA⁺ populations, with cells exhibiting CD45RA expression above 0.7 classified as CD45RA⁺.

(c) Key gene expression profiles of the subclusters were analyzed.

(d) DEG analysis was conducted across three comparisons (A, B, and C) to identify significant transcriptional differences. DEG analysis was restricted to genes expressed in at least 1% of the total population. Genes with a fold change > 2 and q-values < 0.05 were considered significant.

(e) The number of DEGs identified in each comparison was quantified.

(f) DEG quality was assessed by evaluating the frequency of DEG⁺ cells in each subset. The difference in the proportion of DEG⁺ cells between subsets was used as a measure of DEG quality.

(g) Pseudotime of the three clusters.

(h) Frequencies of *C0–9* clusters in healthy individuals (n=4) and patients (n=8).

(i) TCR genes enriched in *C4–C9* clusters.

(j) Clonal diversity and clonality in *C3* and *C4–9* clusters of healthy individuals (n=3) and patients (n=8).

All bar graphs represent mean ± SD, ∗∗p<0.01, ∗p<0.05. ns, non-significant; UMAP, uniform manifold approximation and projection; DEG, differentially expressed genes; TCR, T cell receptor; Tn, naïve T cells; Tcm, central memory T cells; Tem, effector memory T cells; DP, double-positive; DN, double-negative; NSCLC, non-small cell lung cancer; CITE, cellular indexing of transcriptomes and epitopes.

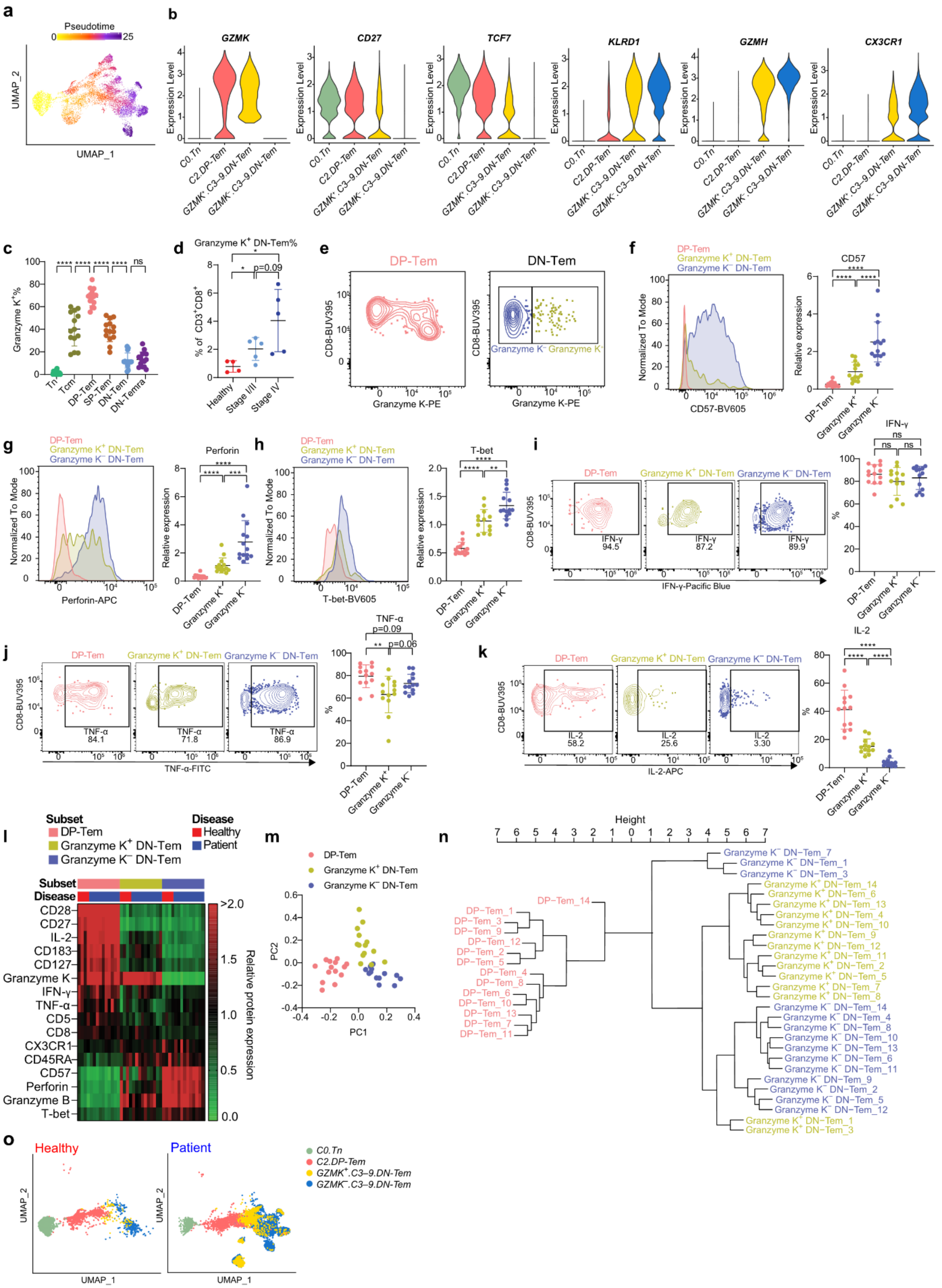

Supplementary Fig. 3. ***GZMK***$^{+}$***.DN-Tem*** **express both early and late effector genes.**

(a) UMAP showing pseudotime analysis.
(b) Violin plot of early (*GZMK*, *CD27*, and *TCF7*) and late (*KLRD1*, *GZMH*, and *CX3CR1*) effector genes.
(c) PBMCs from 4 healthy donors, 5 stage I/II NSCLC patients, and 5 stage IV NSCLC patients were re-stimulated with PMA/ionomycin for 5 hours to assess granzyme K expression. $CD8^+$ T-cell subsets were gated and analyzed for the frequency of granzyme K^+ cells.
(d) The frequency of granzyme K^+ DN-Tem cells was compared across different disease stages.
(e) Gating strategy used to divide DN-Tem cells into Granzyme K^+ and Granzyme K- subsets.
(f–h) Molecular expression levels of (f) CD57, (g) perforin, and (h) T-bet were assessed in three Tem subsets: DP-Tem, granzyme K^+ DN-Tem, and granzyme K- DN-Tem. Relative expression was calculated by dividing the MFI of each subset by the MFI of total Tem cells from the same individual.
(i–k) Frequency of cytokine production in each subset, including (i) IFN-γ, (j) TNF-α, and (k) IL-2.
(l) Heatmap showing molecular expression profiles across the three Tem subsets. Relative expression was calculated by dividing the MFI of each subset by the MFI of total Tem cells from the same individual.
(m–n) Molecular expression data used in the heatmap was further analyzed using (m) principal component analysis and (n) hierarchical clustering to show that granzyme K^+ DN-Tem cells are more closely aligned with granzyme K^- DN-Tem cells rather than DP-Tem cells.
(o) UMAP for healthy individuals and patients after dividing *C3–C9.DN-Tem* into $GZMK^+$ and $GZMK^-$ subsets.
All bar graphs represent mean ± SD, ∗∗∗∗p<0.0001, ∗∗∗p<0.001, ∗∗p<0.01, ∗p<0.05. ns, non-significant; UMAP, uniform manifold approximation and projection; Tn, naïve T cells; Tcm, central memory T cells; Tem, effector memory T cells; DP, double-positive; DN, double-negative; PBMC, peripheral blood mononuclear cell; NSCLC, non-small cell lung cancer; MFI, mean fluorescence intensity; IL, interleukin; IFN, interferon; TNF, tumor necrosis factor.

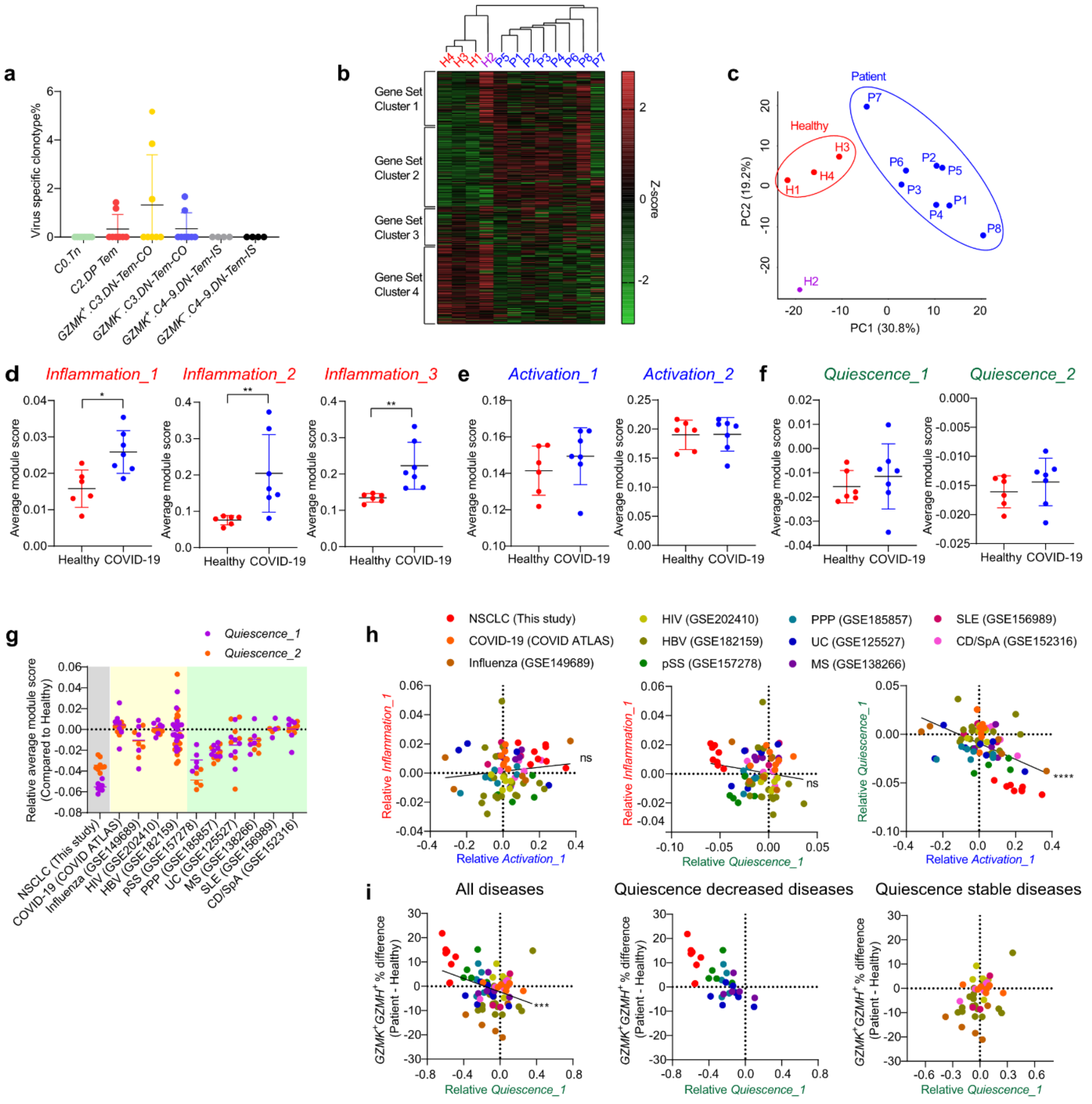

Supplementary Fig. 4. T cell quiescence signatures inversely correlate with T cell activation signatures in peripheral blood CD8⁺ T cells

(a) Frequency of reported virus-specific unique clonotypes in each cluster.
(b) Heat map for normalized average module scores of 644 gene sets in *C2.DP-Tem*. Z-score was color-coded from green (low) to red (high).
(c) PCA plot generated with average module scores of 644 gene sets in *C2.DP-Tem*.
(d–f) Average module scores of indicated gene sets in *DP-Tem* (*GZMK⁺GZMH⁻*) cluster of healthy individuals (n=6) and COVID-19 patients (n=7).
(g) Relative average module score of *Quiescence_1* and *Quiescence_2* gene sets in patients with NSCLC (n=8), COVID-19 (n=7), influenza (n=5), human immunodeficiency virus (HIV; n=6), hepatitis B virus (HBV; n=18), primary Sjögren's syndrome (pSS; n=5), palmoplantar pustulosis (PPP; n=7), ulcerative colitis (UC; n=7), multiple sclerosis (MS; n=5), systemic lupus

erythematosus (SLE; n=3), Crohn's disease and spondyloarthritis (CD and SpA; n=6). Relative average module score was calculated by subtracting average module scores of patients with that of healthy controls from the same dataset in *DP-Tem* (*GZMK*$^+$*GZMH*$^-$) clusters.

(h) Correlation between signature gene sets. Dotted lines represent the average module scores of healthy controls. Solid lines represent linear regression, with p-values calculated using an F-test for non-zero correlation.

(i) Correlation between *Quiescence_1* gene set and proportion of *GZMK*$^+$*GZMH*$^+$ population in CD8$^+$ T cells. Solid lines represent linear regression, with p-values calculated using an F-test for non-zero correlation. Quiescence decreased diseases include NSCLC, pSS, PPP, UC, and MS. Quiescence stable diseases include COVID-19, influenza, HIV, HBV, SLE, and CD/SpA. *GZMK*$^+$*GZMH*$^+$ % difference was measured by subtracting *GZMK*$^+$*GZMH*$^+$ frequency of patients with average of that of healthy controls from the same dataset.

All bar graphs represent mean ± SD. ∗∗∗∗$p<0.0001$, ∗∗$p<0.01$, ∗$p<0.05$. ns, non-significant; Tn, naïve T cells; Tcm, central memory T cells; Tem, effector memory T cells; DP, double-positive; DN, double-negative; PCA, principle component analysis.

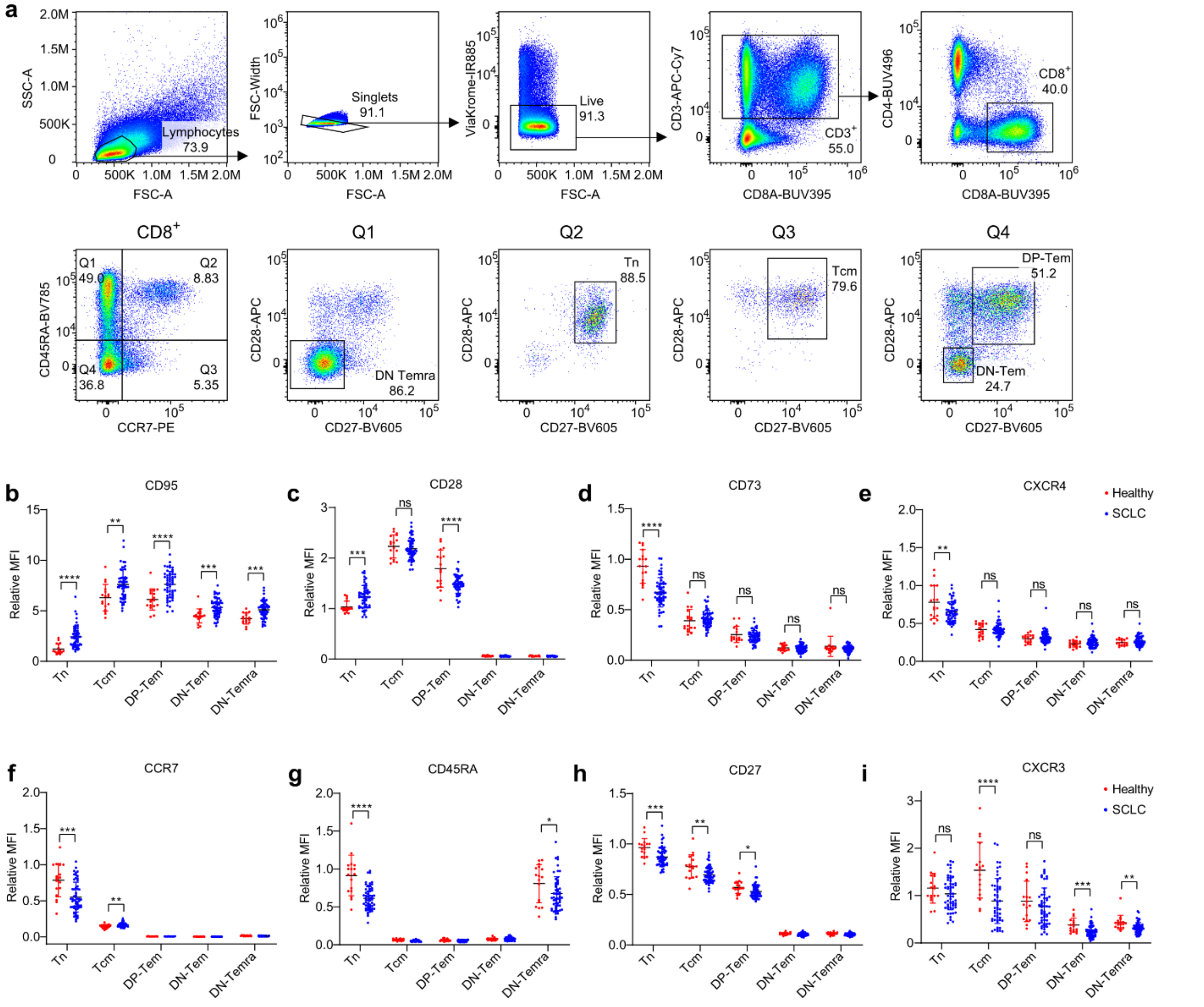

Supplementary Fig. 5. Peripheral blood CD8$^+$ T cells in SCLC patients exhibit gross protein expression alterations.

(a) Gating strategy for CD8$^+$ T cell subsets.

(b–i) Relative MFIs of indicated molecules in each subset from healthy individuals (n=17) and stage ED SCLC patients (n=55). Relative MFI was calculated by dividing MFIs with that of Tn from a healthy individual.

All bar graphs represent mean ± SD. ∗∗∗∗p<0.0001, ∗∗∗p<0.001, ∗∗p<0.01, ∗p<0.05. ns, non-significant; Tn, naïve T cells; Tcm, central memory T cells; Tem, effector memory T cells; Temra, effector memory T cells re-expressing CD45RA; DP, double-positive; DN, double-negative; MFI, mean fluorescent intensity; ED, extensive disease; SCLC, small cell lung cancer.

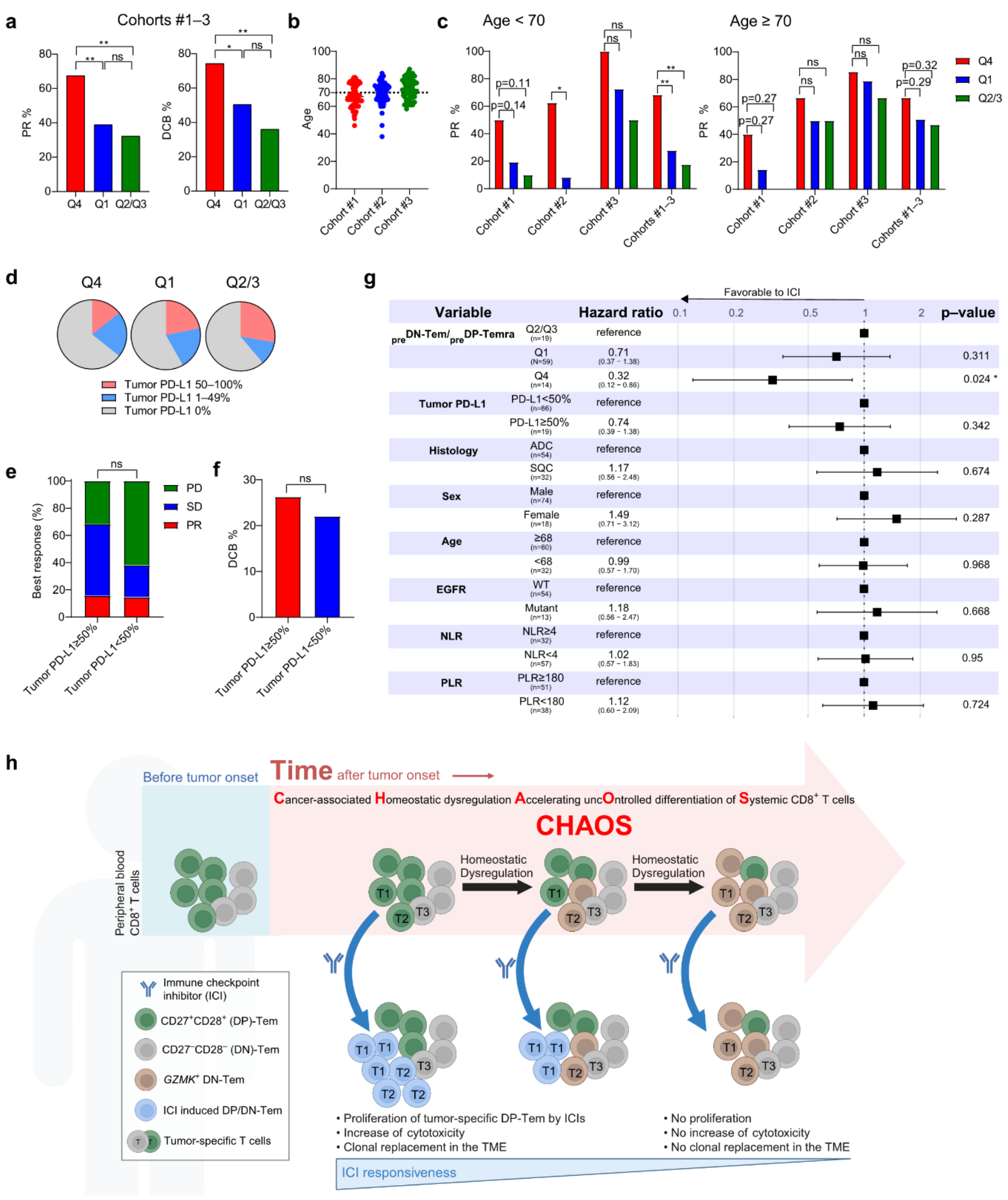

Supplementary Fig. 6. Two subsets of peripheral blood CD8$^+$ T cells predict clinical outcomes to ICI therapy.

(a) Proportion of PR and DCB in Q4, Q1, and Q2/Q3 groups are shown. All patients in the cohorts 1, 2, and 3 are collectively analyzed.

(b) Age distribution of cohorts 1, 2, and 3. Dotted line at age 70 represent the threshold used to divide the patients by age.

(c) Proportion of PR in Q4, Q1, and Q2/Q3 groups after dividing the cohorts by age are shown.

(d) Tumor PD-L1 expression in Q1–Q4 groups in Cohort #1.

(e, f) Relationship between tumor PD-L1 expression and (e) PR and (f) DCB in Cohort #1.
(g) Forrest plot for hazard ratio between PR and indicated variables in Cohort #1. Error bars represent 95% confidence intervals.
(h) Proposed model "Cancer-associated Homeostatic dysregulation Accelerating uncOntrolled differentiation of Systemic $CD8^+$ T cells" (CHAOS). In the earlier times after tumor onset, tumor-specific DP-Tem in the periphery respond to ICI therapy, leading to their proliferation and enhanced cytotoxic activity. These newly activated tumor-specific T cells migrate to the tumor, replenishing the TME. This clonal replacement results in a favorable response to ICI therapy. However, in the later times after tumor onset, CHAOS induces a progressive transition of systemic $CD8^+$ T cells from DP-Tem to DN-Tem (particularly *GZMK*$^+$*.DN-Tem*). Tumor-specific $CD8^+$ T cells that underwent DP-to-DN-Tem transition by CHAOS exhibit impaired responsiveness to ICI therapy, ultimately leading to poor clinical outcomes.
$*p<0.05$. ns, non-significant; ICI, immune checkpoint inhibitor; PD, programmed cell death; PR, partial response; SD, stable disease; PD, progressive disease; DCB, durable clinical benefit; PFS, progression free survival; DP, double-positive; DN, double-negative; Tem, effector memory T cells; Temra, effector memory T cells re-expressing CD45RA; EGFR, epidermal growth factor receptor; NLR, neutrophil-lymphocyte ratio; PLR, platelet-lymphocyte ratio; TME, tumor microenvironment.

**Characteristics**		**Number (n=349)**	**Proportion (%)**
**Age**	30s	2	0.6
	40s	13	3.7
	50s	34	9.7
	60s	137	39.3
	70s	136	39.0
	80s	23	6.6
	NA	4	1.1
**Sex**	Male	288	82.5
	Female	58	16.6
	NA	3	0.9
**Smoking**	Smoker	265	75.9
	Never-smoker	62	17.8
	NA	22	6.3
**Stage**	Stage I NSCLC	33	9.5
	Stage II NSCLC	4	1.1
	Stage III NSCLC	60	17.2
	Stage IV NSCLC	197	56.4
	Stage ED SCLC	55	15.8
**Tumor PD-L1**	0%	136	39.0
	1–49%	60	17.2
	50–100%	68	19.5
	NA	85	24.4
**Histology**	SQC NSCLC	111	31.8
	Non-SQC NSCLC	179	51.3
	SCLC	55	15.8
	NA	4	1.1
**EGFR mutation**	Wildtype	189	54.2
	E19del	12	3.4
	L858R	13	3.7
	S768I	1	0.3
	E19del/T790M	2	0.6
	L858R/T790M	1	0.3
	NA	131	37.5

Supplementary Table 1. Patient information

NA, not available; NSCLC, non-small cell lung cancer; SCLC, small cell lung cancer; LD, limited disease; ED, extensive disease; PD-L1, programmed cell death ligand-1; SQC, squamous cell carcinoma; EGFR, epidermal growth factor.

Characteristics		Number (n=32)	Proportion (%)
**Age**	50s	7	21.9
	60s	17	53.1
	70s	5	15.6
	80s	3	9.4
**Sex**	Male	29	90.6
	Female	3	9.4
**Smoking**	Smoker	28	87.5
	Never-smoker	4	12.5
**Stage**	Stage IV NSCLC	32	100.0
**Tumor PD-L1**	0%	14	43.8
	1–49%	3	9.4
	50–100%	12	37.5
	NA	3	9.4
**Histology**	SQC NSCLC	13	40.6
	Non-SQC NSCLC	19	59.4
**EGFR mutation**	Wildtype	18	56.3
	E19del	3	9.4
	L858R	1	3.1
	NA	10	31.3
**ICI**	Atezolizumab	32	100.0
**IO tx line**	2nd	21	65.6
	3rd	6	18.8
	$>4^{th}$	5	15.6
**Post-therapy collection date**	7	12	37.5
	8	3	9.4
	9	2	6.3
	10	10	31.3
	11	1	3.1
	14	4	12.5
**Best response**	PR	6	18.8
	SD	9	28.1
	PD	16	50.0
	NE	1	3.1

Supplementary Table 2. Patient information

NA, not available; NSCLC, non-small cell lung cancer; PD-L1, programmed cell death ligand-1; SQC, squamous cell carcinoma; EGFR, epidermal growth factor; ICI, immune checkpoint inhibitor; IO, immuno-oncology; tx, therapy; PR, partial response; SD, stable disease; PD, progressive disease; NE, inevaluable.

Patient	P1	P2	P3	P4	P5	P6	P7	P8
**Age**	72	64	66	58	67	62	44	66
**Sex**	M	M	M	F	M	M	M	M
**Smoking**	Smoker	Smoker	Smoker	Never	Smoker	Smoker	Smoker	Smoker
**Stage**	IB	IA3	IB	IA2	IVB	IVB	IVB	IVA
**Histology**	ADC	ADC	SQC	ADC	ADC	ADC	ADC	ADC
**EGFR mutation**	L858R	WT	WT	L858R	WT	WT	WT	L858R
**Tumor PD-L1**	90	100	5	0	50	80	15	60

Supplementary Table 3. Patient information for scRNA-seq

ADC, adenocarcinoma; SQC, squamous cell carcinoma; EGFR, epidermal growth factor; WT, wildtype; PD-L1, programmed cell death ligand-1.

Characteristics		Number (n=70)	Proportion (%)
**Age**	40s	1	1.4
	50s	9	12.9
	60s	37	52.9
	70s	19	27.1
	80s	4	5.7
**Sex**	Male	66	94.3
	Female	4	5.7
**Smoking**	Smoker	64	78.3
	Never-smoker	6	21.7
**Stage**	Stage IV NSCLC	70	100.0
**Tumor PD-L1**	0%	37	52.9
	1–49%	16	22.9
	50–100%	16	22.9
	NA	1	1.4
**Histology**	SQC NSCLC	29	41.4
	Non-SQC NSCLC	41	58.6
**EGFR mutation**	Wildtype	45	64.3
	E19del	2	2.9
	L858R	1	1.4
	S768I	1	1.4
	NA	21	30.0
**Therapy**	Atezolizumab	70	100.0
**IO tx line**	2nd	54	77.1
	3rd	16	22.9
**Best response**	PR	14	20.0
	SD	22	31.4
	PD	34	48.6
**Durable benefit**	DCB	21	30.0
	NCB	48	68.6
	NA	1	1.4
**PFS**	0–5 months	48	68.6
	5–10 months	11	15.7
	10–15 months	4	5.7
	15–20 months	1	1.4
	20–25 months	5	7.1
	NA	1	1.4

Supplementary Table 4. Patient information for Cohort #1

NA, not available; NSCLC, non-small cell lung cancer; PD-L1, programmed cell death ligand-1; SQC, squamous cell carcinoma; EGFR, epidermal growth factor; IO, immuno-oncology; tx, therapy; PR, partial response; SD, stable disease; PD, progressive disease; DCB, durable clinical benefit; NCB, no durable clinical benefit; PFS, progression free survival.

Characteristics		Number (n=54)	Proportion (%)
**Age**	30s	1	1.9
	40s	1	1.9
	50s	1	1.9
	60s	20	37.0
	70s	27	50.0
	80s	4	7.4
**Sex**	Male	50	92.6
	Female	4	7.4
**Smoking**	Smoker	45	83.3
	Never-smoker	9	16.7
**Stage**	Stage IV NSCLC	54	100.0
**Tumor PD-L1**	0%	26	48.1
	1–49%	16	29.6
	50–100%	11	20.4
	NA	1	1.9
**Histology**	SQC NSCLC	23	42.6
	Non-SQC NSCLC	31	57.4
**EGFR mutation**	WT	36	66.7
	NA	18	33.3
**Therapy**	K	6	11.1
	KAP	28	51.9
	KTC	20	37.0
**IO tx line**	1st	54	100.0
**Best response**	PR	23	42.6
	SD	15	27.8
	PD	16	29.6
**Durable benefit**	DCB	24	44.4
	NCB	30	55.6

Supplementary Table 5. Patient information for Cohort #2

NA, not available; NSCLC, non-small cell lung cancer; PD-L1, programmed cell death ligand-1; SQC, squamous cell carcinoma; EGFR, epidermal growth factor; K, pembrolizumab alone; KAP, pembrolizumab, pemetrexed, and cisplatin combination therapy; KTC, pembrolizumab, paclitaxel, and carboplatin combination therapy; IO, immuno-oncology; tx, therapy; PR, partial response; SD, stable disease; PD, progressive disease; DCB, durable clinical benefit; NCB, no durable clinical benefit.

**Characteristics**		**Number (n=55)**	**Proportion (%)**
**Age**	50s	1	1.8
	60s	19	34.5
	70s	25	45.5
	80s	10	18.2
**Sex**	Male	52	94.5
	Female	3	5.5
**Smoking**	Smoker	48	87.3
	Never-smoker	7	12.7
**Stage**	Stage ED SCLC	55	100.0
**Histology**	SCLC	55	100.0
**Therapy**	ACE	55	100.0
**IO tx line**	1st	55	100.0
**Best response**	CR	1	1.8
	PR	41	74.5
	SD	6	10.9
	PD	5	9.1
	NA	2	3.6
**Durable benefit**	DCB	43	78.2
	NCB	12	21.8

Supplementary Table 6. Patient information for Cohort #3

NA, not available; ED, extensive disease; SCLC, small cell lung cancer; ACE, atezolizumab, carboplatin, and etoposide combination therapy; IO, immuno-oncology; tx, therapy; CR, complete response; PR, partial response; SD, stable disease; PD, progressive disease; DCB, durable clinical benefit; NCB, no durable clinical benefit.

Characteristics		Number (n=45)	Proportion (%)
**Age**	50s	7	15.6
	60s	16	35.6
	70s	21	46.7
	80s	1	2.2
**Sex**	Male	40	88.9
	Female	5	11.1
**Smoking**	Smoker	41	91.1
	Never-smoker	4	8.9
**Stage**	Stage III NSCLC	45	100.0
**Tumor PD-L1**	0%	18	40.0
	1–49%	15	33.3
	50–100%	12	26.7
**Histology**	SQC NSCLC	26	57.8
	Non-SQC NSCLC	19	42.2
**EGFR mutation**	WT	19	42.2
	L858R	2	4.4
	NA	24	53.3
**1st line therapy**	CCRT	45	100.0
**Best response**	PR	28	62.2
	SD	14	31.1
	PD	3	6.7
**Consolidation**	Durvalumab	19	42.2
	None	26	57.8
**PFS**	0–5 months	6	13.3
	5–10 months	12	26.7
	10–15 months	7	15.6
	15–20 months	5	11.1
	20–25 months	6	13.3
	25–30 months	8	17.8
	NA	1	2.2

Supplementary Table 7. Patient information for Cohort #4

NA, not available; NSCLC, non-small cell lung cancer; PD-L1, programmed cell death ligand-1; SQC, squamous cell carcinoma; EGFR, epidermal growth factor; CRT, concurrent chemoradiation therapy; PR, partial response; SD, stable disease; PD, progressive disease; PFS, progression free survival.
